# N-Alkylation through the Borrowing Hydrogen Pathway Catalyzed by the Metal–Organic Framework-Supported Iridium–Monophosphine Complex

**DOI:** 10.1021/acsami.4c02143

**Published:** 2024-04-17

**Authors:** Wenmiao Chen, Muhammad Sohail, Yempally Veeranna, Yihao Yang, Ashfaq A. Bengali, Hong-Cai Zhou, Sherzod T. Madrahimov

**Affiliations:** † Department of Arts and Science, Texas A&M University at Qatar, Education City, Post Office Box 23874, Doha, Qatar; ‡ Department of Chemistry, Texas A&M University, College Station, Texas 77843-3255, United States; § School of Materials Science and Engineering, China University of Petroleum (East China) Qingdao, Shandong 266580, People’s Republic of China; ∥ Department of Natural Sciences, Faculty of Science and Engineering, Manchester Metropolitan University, Manchester M15 6BH, United Kingdom

**Keywords:** metal−organic frameworks, heterogeneous catalysis, N-alkylation, borrowing hydrogen, pharmaceutical precursor synthesis

## Abstract

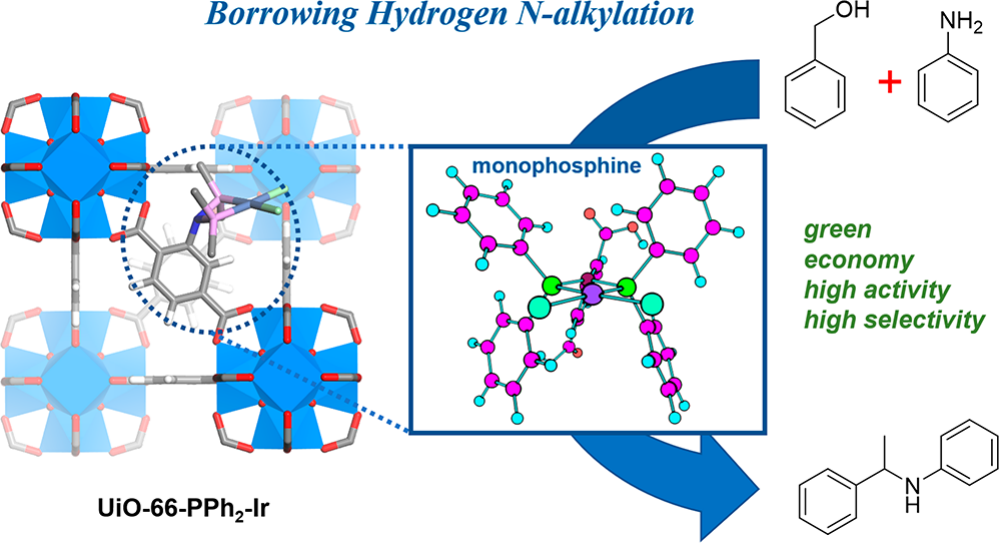

Further development in the area of medicinal chemistry requires facile and atom-economical C–N bond formation from readily accessible precursors using recyclable and reusable catalysts with low process toxicity. In this work, direct N-alkylation of amines with alcohols is performed with a series of Ir–phosphine-functionalized metal–organic framework (MOF) heterogeneous catalysts. The grafted monophosphine–Ir complexes were studied comprehensively to illustrate the ligand-dependent reactivity. The afforded MOF catalysts exhibited high reactivity and selectivity toward *N*-alkylamine product formation, especially UiO-66–PPh_2_–Ir, which showed 90% conversion after recycling with no catalyst residue remaining in the product after the reaction. Furthermore, analyses of the active catalyst, mechanistic studies, control experiments, and H_2_ adsorption tests are consistent with the conclusion that immobilization of the iridium complex on the MOF support enables the formation of the iridium–monophosphine complex and enhances its stability during the reaction. To illustrate the potential of the catalyst for application in medicinal chemistry, two pharmaceutical precursors were synthesized with up to 99% conversion and selectivity.

## 1 Introduction

C–N bond formation reactions, such as the Ullmann reaction,^1^ Buchwald–Hartwig coupling reaction,^2^ hydroamination,^3^ and reductive amination,^4^ are widely utilized in synthesis of reactive intermediates in pharmaceuticals, agrochemicals, surfactants, and natural product derivatives. This is usually done through the cross coupling of halides or pseudohalides with amines.^5^ In comparison to traditional alkyl halide-initiated reaction pathways, N-alkylation of primary amines with alcohols is more advantageous because of the lower toxicity and wider availability of alcohol substrates over halogenated organics, because organic halides are usually made from halogenation of alcohols in industry.^6−10^ In addition, H_2_O is the only biproduct in N-alkylation with alcohols, making it an atom-economical and greener alternative to reactions involving alkyl halides. Traditionally, the most active homogeneous catalysts [turnover number (TON) > 500] are based on noble metals (Ru,^11,12^ Rh,^13^ Pd,^14^ and Ir^15^), with potentially limited applicability as a result of their high cost and inefficient recyclability. Beginning in the early 2000s, research efforts on further developing this reaction have seen a resurgence with increasing reports in the literature for both homo- and heterogeneous catalysis.^16^ However, their application in pharmaceutical synthesis presents some important drawbacks, mainly related to the low recyclability of expensive catalysts and/or unavoidable catalyst residue in the product.

Heterogenization of organometallic complexes while maintaining high reactivity and selectivity is a promising strategy for catalyst development that enables combining the favorable characteristics of homo- and heterogeneous reactions. In comparison to a nanoparticle^17^ or immobilized metallic compounds,^18^ heterogenized complexes help achieve site isolation and enable facile electronic and steric optimization of the immobilized catalyst.^19^ However, traditional heterogeneous supports, such as porous carbon,^20^ zeolites,^21^ and metal oxides,^22^ are not readily amenable for rational molecular tuning to afford specially designed coordination of the active center.

Metal–organic frameworks (MOFs), an emerging type of porous crystalline materials with organic ligands and inorganic metal clusters, enable orderly arrangement of active sites with uniform distribution and high accessibility toward substrates.^23^ Moreover, through rational design, the ligands immobilized on MOFs are able to provide unique coordination environments that lead to superior catalytic activities and tailorable selectivities.^24−26^ Because of these unique advantages, MOF-supported molecular complexes have increasingly been applied to promote a variety of catalytic transformations.^27^ This includes reports from our group on both surface-anchored and pore-confined MOF-immobilized catalysts for recyclable C–C coupling reactions.^25,28^ Also, previous work has shown that specific coordination modes can easily be achieved through postsynthetic rearrangement of the ligands within the MOF scaffold.^24,29^ Furthermore, isolated complexes supported on MOFs prevent deactivation pathways like dimerization and agglomeration, leading to greater stability.^30,31^ Phosphine ligands are widely exploited, strong-field auxiliary ligands for organometallic catalysts.^32−34^ For example, replacement of ancillary nitrogen ligands with their phosphine analogues not only allows the reaction to operate under milder conditions but enables asymmetric amination with a chiral phosphoric acid.^35,36^ Thus, Ir–phosphine complexes supported on MOFs are good candidates for the design of highly effective and recyclable catalysts for the N-alkylation reaction.

In this work, we report the synthesis of monodentate complexes of iridium with a phosphine or phosphinoamine ligand immobilized on the surface of nanosized (20 nm) UiO-66 MOF. In addition, we demonstrate the ability of these complexes to promote the C–N coupling reaction between alcohols and amines by the borrowing hydrogenation pathway. The reported chemistry is very general and applicable toward a wide variety of alcohols and amines. Reaction progression was monitored by time-resolved infrared (IR) spectroscopy, and the carbonyl-containing intermediates were observed, supporting the borrowing hydrogenation pathway. Lastly, the application of this catalyst toward the synthesis of a natural product and a pharmaceutically active compound is demonstrated. This work highlights the applicability of immobilized monophosphine complexes for N-alkylation of alcohols and paves the way for the rational design of MOF heterogenized catalysts for pharmaceutical applications.

## 2 Experimental Section

### 2.1 Synthesis of UiO-66

UiO-66 with a nanosize was synthesized according to a previous procedure published by Morris et al.^37^ Benzene-1,4-dicarboxylic acid (500 mg, 2.4 mmol) was dissolved in 30 mL of *N*,*N*-dimethylformamide (DMF). In a separate vial, zirconyl chloride octahydrate (420 mg, 1.32 mmol) was dissolved in 30 mL of DMF. After sonication, the solutions were combined, and 6 mL of acetic acid was added and further sonicated for 15 min. The combined solution was heated in a temperature-controlled oven at 90 °C for 18 h. Then, the white jelly-like MOF nanoparticle was purified by centrifugation at 6000 rpm for 20 min followed by solvent exchange (3× DMF and 3× acetone) over a 24 h period to afford white MOF powder. The nanoparticles were weighted and collected with the yield of 73% (calculated from ZrCl_4_).

### 2.2 Synthesis of the Mono(diphenylphosphino)amine Ligand

The BDC–NHPPh_2_ ligand was synthesized according to the literature, with slight modification.^38^ In a 250 mL flask, dimethyl aminoterephthalic acid (1.04 g, 5 mmol) and triethyl amine (2 g, 20 mmol) were dissolved in 50 mL of dichloromethane. Then, the reaction was cooled to 0 °C in an ice bath. To the resulting suspension was added chlorodiphenylphosphine (2.2 g, 10 mmol) dropwise over 15 min. Then, the reaction was stirred at room temperature under N_2_ for 12 h. The solution was dried through rotary evaporation and then neutralized with 10% HCl aqueous solution, and the product was collected by filtration (1.77 g, 90% yield). The ester ligand (0.80 g, 2.03 mmol) was further hydrolyzed in a 30 mL solution of a THF/MeOH/H_2_O mixture (1:1:1) with 0.77 g of KOH (12.5 mmol) at room temperature for 16 h. Then, the solvent was removed through rotary evaporation, cooled at 0 °C, and neutralized with 2 M HCl. The yellow solid was filtered and washed with deionized water and methanol to obtain a light yellow solid (90% yield).

### 2.3 Synthesis of UiO-66–PPh_2_/NHPPh_2_

UiO-66–PPh_2_ was synthesized using 400 mg of UiO-66 and 250 mg of 2-(diphenylphosphino)terephthalic acid in a vial. A total of 25 mL of DMF was added to form a solution mixture and was stirred overnight. The solution was then washed with DMF twice. A total of 20 mL of DMF and 2 mL of HCl were added to remove the unattached or end-attached ligands. Finally, it was dried in a vacuum overnight. A total of 220 mg of UiO-66–PPh_2_ was obtained. To evaluate the exchange ratio, 3 mg of the MOF sample was dissolved in 0.5 mL of deuterated dimethyl sulfoxide (DMSO-*d*_6_) with D_2_SO_4_ (5 drops), which was then analyzed by ^31^P nuclear magnetic resonance (NMR) spectroscopy. The molecular weights of UiO-66–PPh_2_/NHPPh_2_ based on immobilized ligands were calculated by the ratio of the phosphine peak coming from the MOF solution against the ^31^P peak of the phosphonic acid D_2_O solution of a known concentration (0.01 mol/L, chemical shift at 0 ppm) added as an external standard in a capillary tube.

### 2.4 Synthesis of UiO-66–PPh_2_–Ir/NHPPh_2_–Ir

To a mixture of UiO-66–PPh_2_ (100 mg) and chloro(1,5-cyclooctadiene)iridium(I) dimer (50 mg) was added 10 mL of DMF into the vial and mixed uniformly at room temperature overnight. Upon completion of the reaction, the resulting MOF was washed several times with DMF until the liquid on top became colorless. Finally, the UiO-66–PPh_2_–Ir catalyst obtained was weighed and stored in a vial as a dark brown powder.

### 2.5 Catalytic Activity of UiO-66–PPh_2_–Ir

In a typical run of a N-alkylation reaction optimization test, 0.3 mmol of benzyl alcohol (29 μL), 0.2 mmol of aniline (18 μL) and 0.4 mmol of base were combined in a vial containing 1 mL of chosen solvent under argon gas. With a total of 2–3 mg of the synthesized catalyst (1 mol %), UiO-66–PPh_2_–Ir was then added to the solution. The vial was incubated at 110 °C for 12 h, followed by centrifugation to separate the solid. After the reaction, 0.2 mmol of mesitylene (28 uL) was added as an internal standard and the conversion and yield were analyzed by gas chromatography–flame ionization detection (GC–FID). Substrate screening was performed in 1 mL dioxane under the same conditions. The product was analyzed by NMR spectra in chloroform-*d*_3_.

## 3 Results and Discussion

### 3.1 Synthesis and Characterization of the MOF Catalyst

**Scheme 1:**
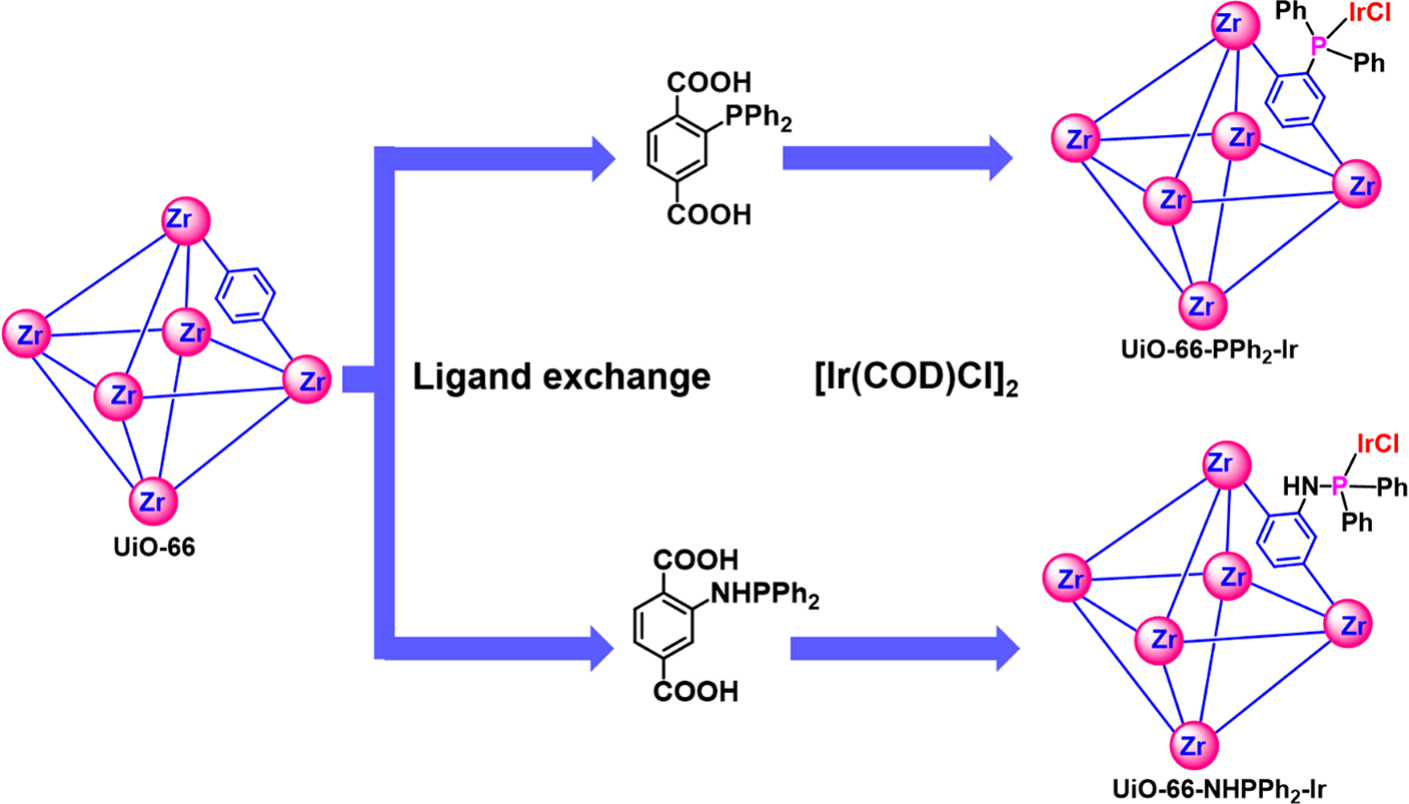
Design and Fabrication of Phosphine Ir-Immobilized UiO-66–PPh_2_–Ir and Bidentate UiO-66–NHPPh_2_–Ir

**Figure 1:**
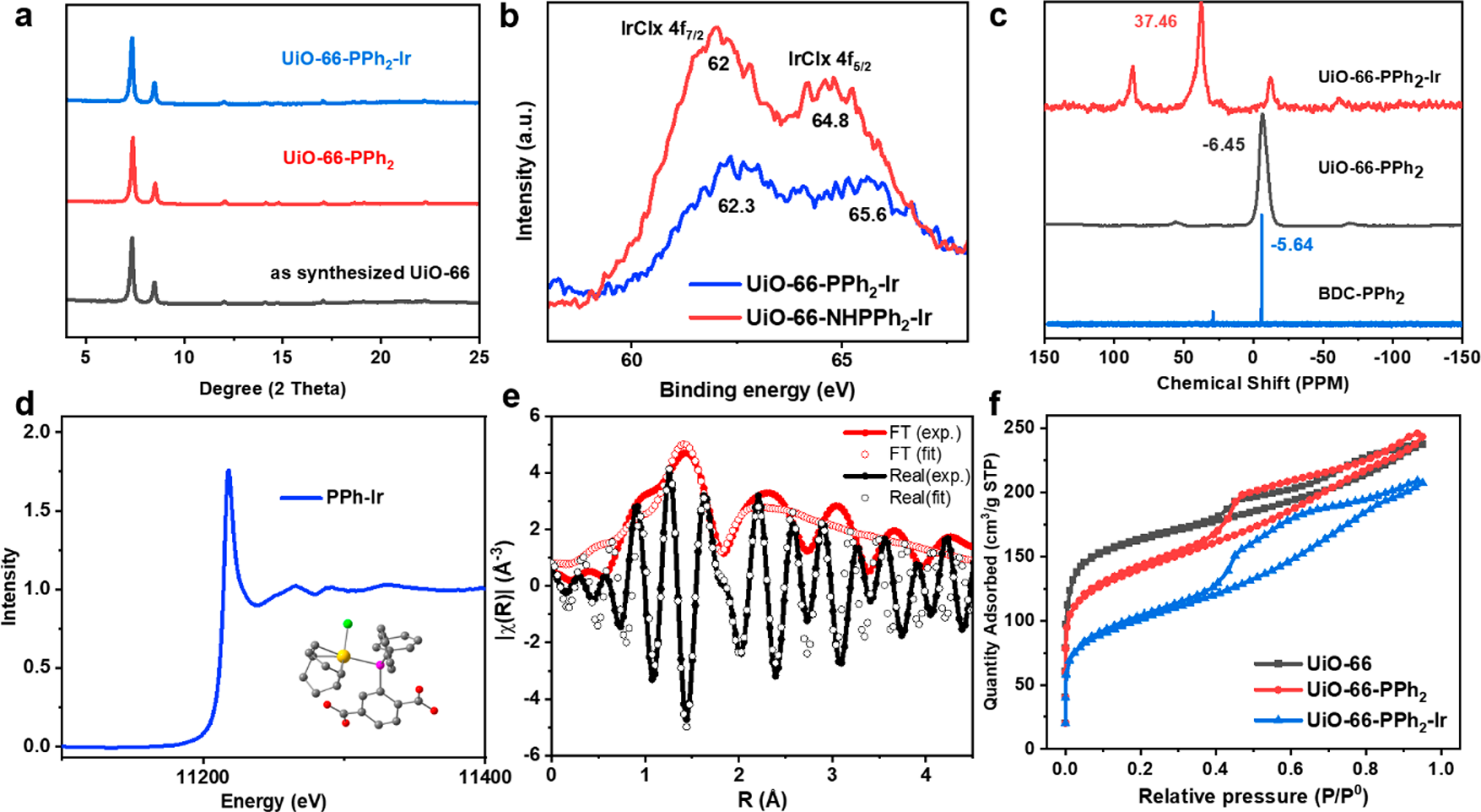
Images of (a) PXRD of UiO-66, UiO-66–PPh_2_, and UiO-66–PPh_2_–Ir. (b) XPS analysis of UiO-66–PPh_2_–Ir and UiO-66–NHPPh_2_–Ir. (c) ^31^P SSNMR spectra of UiO-66–PPh_2_ and UiO-66–PPh_2_–Ir and ^31^P NMR of BDC–PPh_2_ in DMSO–*d*_6_. (d) XANES spectra of the Ir L_3_ edge on UiO-66–PPh_2_–Ir. The structure of BDC–PPh_2_–Ir is shown in the inset (hydrogens are deleted for clarity). (e) EXAFS fitting of Ir in UiO-66–NHPPh_2_–Ir in *R* space containing both Fourier transform and real components. (f) N_2_ sorption isotherms UiO-66, UiO-66–PPh_2_, and UiO-66–PPh_2_–Ir.

The MOF catalysts were prepared through a two-step postsynthetic ligand exchange and metalation (Scheme 1) process. The pristine UiO-66 nanoparticles were made in gram-scale quantities following a previously reported procedure^28^ (section 2.1) with an average size of 20 nm (Figure S1 of the Supporting Information). Diphenyl phosphine terephthalic acid (BDC–PPh_2_) is commercially available, while monophosphinoamine (BDC–NHPPh_2_) was synthesized through an aminolysis reaction between the methyl ester of aminoterephthalic acid and chlorodiphenyl phosphine in the presence of triethylamine followed by ester hydrolysis based on a previously reported procedure^39^ (section 2.2). To confirm the structure of BDC–NHPPh_2_, a crystal structure of the synthesized methyl ester was obtained through single-crystal X-ray diffraction (SCXRD), and the data are summarized in Table S1 of the Supporting Information. The phosphine ligands were incorporated into the MOF structure through a well-established postsynthetic ligand exchange method (section 2.3). Following the phosphine exchange, MOF crystals with immobilized phosphine ligands were metalated by the [Ir(COD)Cl]_2_ (COD = 1,5-cyclooctadiene) precursor in methanol solution under inert gas to afford UiO-66–PPh_2_–**Ir** (section 2.4). Powder X-ray diffraction (PXRD) before and after functionalization were almost identical, demonstrating the retention of the framework crystallinity through postsynthetic ligand exchange and metalation (Figure 1a and Figure S2 of the Supporting Information). The incorporation of each of the corresponding ligands to form UiO-66–PPh_2_/NHPPh_2_ was verified by matching ^1^H and ^31^P NMR spectra obtained from MOF samples digested in D_2_SO_4_/DMSO-*d*_6_ to the spectra of independently synthesized acids (Figures S3–S6 of the Supporting Information). The extent of ligand immobilization in the UiO-66–PPh_2_/NHPPh_2_ samples was quantified using an external standard with a known concentration in a capillary tube placed inside a NMR tube with the digested sample (section 2.3 and Table S2 of the Supporting Information). Accordingly, the calculated exchange ratio of the phosphine-functionalized linkers, BDC–PPh_2_/NHPPh_2_, to the total amount of linkers in the MOF is 1:4. This can be represented by the general formula Zr_6_O_4_(OH)_4_–(BDC)_4.5_–(BDC–PPh_2_/NHPPh_2_)_1.5_ (Table S2 of the Supporting Information).

To confirm the coordination environment of the metal, the metalated MOFs were first characterized by X-ray photoelectron spectroscopy (XPS) (Figure 1b), where the binding energy of Ir on UiO-66–PPh_2_–Ir was 62.3 and 65.6 eV, matching the monophosphine coordinated Ir–P complex.^40,41^ In contrast, UiO-66–NHPPh_2_–Ir exhibits lower binding energies of 62.0 and 64.8 eV, stemming from the slightly lower valence of Ir with weaker back donation from metal to aminophosphine. Furthermore, no XPS peaks of [Ir(COD)Cl]_2_ were found at 63.2 and 66.2 eV, indicating no residue of unreacted precursor in the MOF samples. The Ir–Zr molar ratio measured by inductively coupled plasma (ICP) was 1:6, which is slightly lower than the ratio of ligands, yielding the formula Zr_6_O_4_(OH)_4_–(BDC)_4.5_–(BDC–PR_2_)_1.5_–Ir_1.0_ (Table S3 of the Supporting Information). Energy-dispersive spectroscopy (EDS) elemental mapping data show an even distribution of Ir and P throughout the MOF surface in agreement with the formation of immobilized molecular catalysts (Figure S7 of the Supporting Information). Furthermore, ligand exchange on MOF samples was probed by ^31^P solid-state NMR (SSNMR) of UiO-66–PPh_2_ and referenced to the spectrum of homogeneous BDC–PPh_2_ (Figure 1c). Upon binding of Ir to form the immobilized monophopshine complex, the ^31^P peak shifts from about −6 ppm in UiO-66–PPh_2_ to 37 ppm in UiO-66–PPh_2_–Ir (Figure 1c). X-ray absorption spectroscopy (XAS) was conducted with the aim of probing the electronic structure and the local atomic environment of Ir on the MOF. The formation of the monophosphine–Ir complex was further supported by the white line X-ray absorption near edge structure (XANES) of Ir at the L_3_ edge, using the BDC–PPh_2_–Ir complex as a structural model (Figure 1d). Extended X-ray absorption fine structure (EXAFS) data fitted by Fourier transform revealed that Ir had a tetratopic coordination with P and Cl and a COD molecule (Figure 1e). The fitted Ir–P, Ir–C, and Ir–Cl bond lengths are 2.20, 2.45, and 2.57 Å, respectively, which correspond to the literature and theoretical model.^40^ The fitting parameters as well as *k*^3^-weighted EXAFS data are summarized in Figure S8 and Table S4 of the Supporting Information. The surface ligand functionalization was further supported by the N_2_ adsorption isotherm and pore size distribution (Figure 1f and Figure S9 of the Supporting Information). After ligand exchange, the Brunauer–Emmett–Teller (BET) surface area decreases from 603.5 to 500.2 m^2^/g for UiO-66 and UiO-66–PPh_2_, respectively. Postsynthetic metalation further decreases the surface area to 356.5 m^2^/g, which originates from the high molar mass of Ir. The pore size distribution supports the unchanged pore environment, in which the mesopores are inherited from defective nano-UiO-66 and slightly increase following postsynthetic treatment. The defects and mesopores are reported to enhance the mass transfer and overall activity of the catalysts.^42−44^ The effect of the MOF scaffold was further supported by the H_2_ adsorption isotherms (Figure S10 of the Supporting Information), where UiO-66–PPh_2_–Ir showed higher hydrogen adsorption than UiO-66–PPh_2_ and even higher hydrogen adsorption than UiO-66 at a low pressure range (<20 *P*/*P*^0^). This high affinity for hydrogen is ascribed to the activation of H_2_ by the Ir center, which is exploited in this work and numerous previous reports.^45−47^

### 3.2 N-Alkylation Activity

To evaluate the catalytic activity toward the N-alkylation reaction, a model reaction between aniline and benzyl alcohol was conducted. The reactions were catalyzed by 3 mg (0.6 mol % Ir) loading of UiO-66–PPh_2_–Ir. Specifically, optimization of the catalytic activity with respect to the solvent, choice of base, and temperature was performed (see entries 1–6 in Table S5 of the Supporting Information). The optimized condition using KO*t*Bu as a base at 110 °C in dioxane resulted in 95% conversion and 86% selectivity for the targeted amine product in 12 h (entry 4 in Table S5 of the Supporting Information). The 86% selectivity represents the selectivity for the amine product over the non-hydrogenated imine product that forms via condensation of amine and carbonyl formed through initial dehydrogenation of the alcohol. To demonstrate the necessity of every component of the catalytic system for catalytic activity, we have also conducted the background reactions: the combination of non-functionalized UiO-66 with [IrCODCl]_2_ showed no reaction (entry 7 in Table S5 of the Supporting Information). UiO-66–PPh_2_ without any iridium showed no reaction as well (entry 8 in Table S5 of the Supporting Information), indicating the vital role of the Ir–P complex. The 1:1 molar ratio of BDC–PPh_2_/[IrCODCl]_2_ solution, which represents the homogeneous analogue of the reaction, provided 94% conversion; however, the selectivity for the targeted amine product was only 23% (entry 9 in Table S5 of the Supporting Information). This low selectivity is possibly due to the unstable nature of the homogeneous complex, which is depleted before the full imine rehydrogenation can be achieved, and formation of complexes with inferior activity, such as the Ir–phosphine dimer. The observed activity of the immobilized complex is comparable to those observed for preactivated homogeneous ligand–Ir complexes.^15^ Through variation of the composition of the immobilized catalyst (metal, MOF, and ligand), we evaluated the influence of these components on the reaction. Exchange of Ir to Rh results in UiO-66–PPh_2_–Rh, with much lower conversion of 78% (entry 10 in Table S5 of the Supporting Information). At the same time, we changed the metal of UiO-66 to hafnium, using UiO-66–Hf–PPh_2_–Ir for the reaction. Accordingly, we observed no significant change in the conversion or selectivity of the reaction (entry 11 in Table S5 of the Supporting Information). Using UiO-66–NHPPh_2_–Ir as a catalyst leads to conversion of only 65%, which may be ascribed to the relatively poor electron-donating ability of the phosphineamine ligand, as mentioned above (entry 12 in Table S5 of the Supporting Information). The role of the base is to deprotonate the alcohol and preactivate the catalyst in the first catalytic cycle of the reaction, and reducing the base loading to 0.1 equiv did not influence the conversion or selectivity (entry 13 in Table S5 of the Supporting Information). The heterogeneous nature of the catalyst was also confirmed by the “hot filtration test”, which showed that the reaction stopped at 53% conversion following the removal of the catalyst after 2 h of the MOF-catalyzed reaction (Figure S11 of the Supporting Information), confirming the heterogeneous nature of the catalyst. Moreover, the ICP measurement of the reaction solution as well as the recovered catalyst after the reaction showed no evidence of Ir leaching, and the Ir/Zr atomic ratio remained almost unchanged (0.153) for the recovered catalyst compared to the fresh catalyst (Table S3 of the Supporting Information). The catalyst maintained its activity and selectivity for at least 4 cycles of reaction/catalyst recovery, displaying both conversion and selectivity of above 90% (Figure S12 of the Supporting Information). This leads to an overall TON of 330 for UiO-66–PPh_2_–Ir, which indicates high reaction efficiency among all reported heterogeneous catalysts.^48^ The recycled catalyst also retains its crystallinity according to PXRD, indicating good stability and recyclability of the MOF-supported catalysts (Figure S13 of the Supporting Information).

The systematic study of the generality of UiO-66–PPh_2_–Ir was conducted under optimized conditions (Table 1). UiO-66–PPh_2_–Ir displayed high conversions and selectivities for the N-alkylation reactions of aniline with a series of alkyl alcohol substrates with a range of electron-withdrawing or -donating substituents (Table 1). The observed isolated conversions of the coupling products were all excellent (over 90%) regardless of the type of alcohol. In addition to the benzylic alcohols, the catalyst was also effective for aliphatic alcohols with varying electronic and structural properties. For example, we observed similarly high isolated yields for aliphatic alcohols with electron-donating substituents, *n*-propanol (**1c**), isopropanol (**1d**), and isobutanol (**1e**), as well as for alcohols with electron-withdrawing groups, such as trifluoromethyl (**1b**). Moreover, a secondary alcohol, benzhydrol (**1i**), was tested to evaluate the steric effect and showed 94% conversion with 95% selectivity for the amine product. The tolerance for sterically hindered substrates likely originates from the surface-grafted nature of the active catalyst, which shows no confinement effect like other MOF-immobilized systems.^25,49^ The observed high activity for a variety of alcohols based on the observations from the homogeneous analogues of this reaction likely comes from the strong electron-donating effect of the monophosphine ligand.^8,45^ Similarly high conversions and selectivities were also observed for the reactions of benzyl alcohol with a number of alkyl and aryl amines with varying electronic and structural properties (**1j**–**1p**). It is noteworthy to mention that the steric effect of the substrate was not dominant because the *ortho*-substituted aromatic amines (OMe, **1m**) or (Br, **1o**) showed comparable reactivity with the *para*-substituted substrates.

**Table 1:**
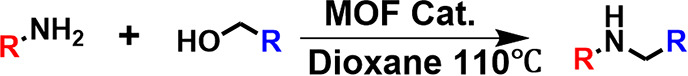

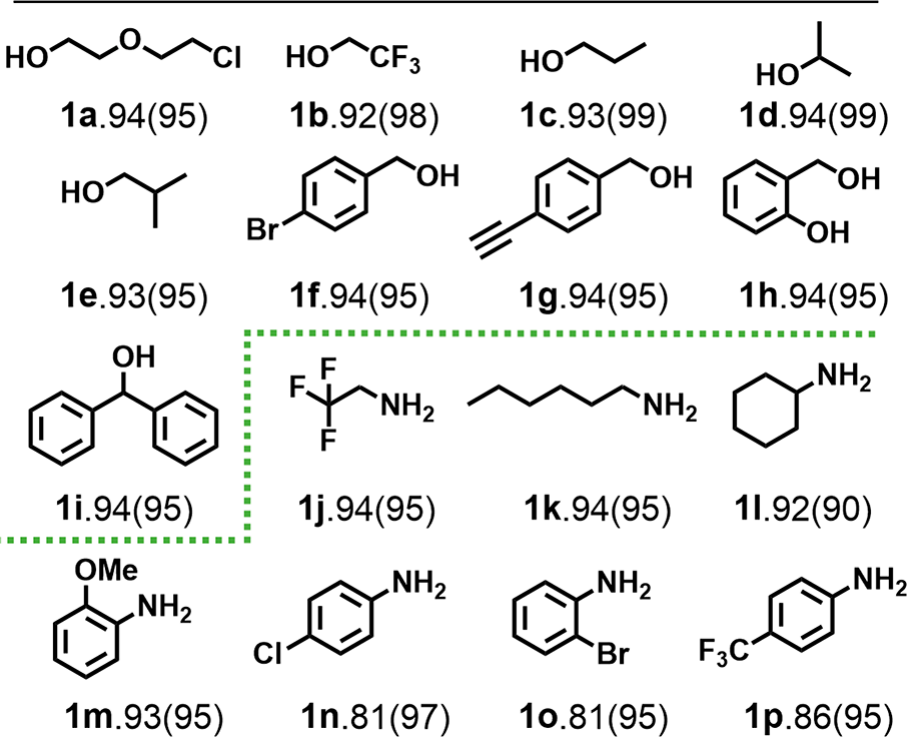
Substrate Screening of the N-Alkylation Reaction^a^^,^^b^^,^^c^

a Reaction conditions: aniline (0.2 mmol), benzyl alcohol (0.3 mmol), dioxane (2 mL), KO*t*Bu (0.4 mmol), time (12 h), and MOF (1 mol %).

b Conversion and selectivity were obtained from NMR with mesitylene as internal standard (Supporting S2.5).

c Substrate screening: 1a-1i are various alcohol reacted with aniline; 1j-1p are various amines reacted with benzyl alcohol, separated by the green dot line.

**Scheme 2:**
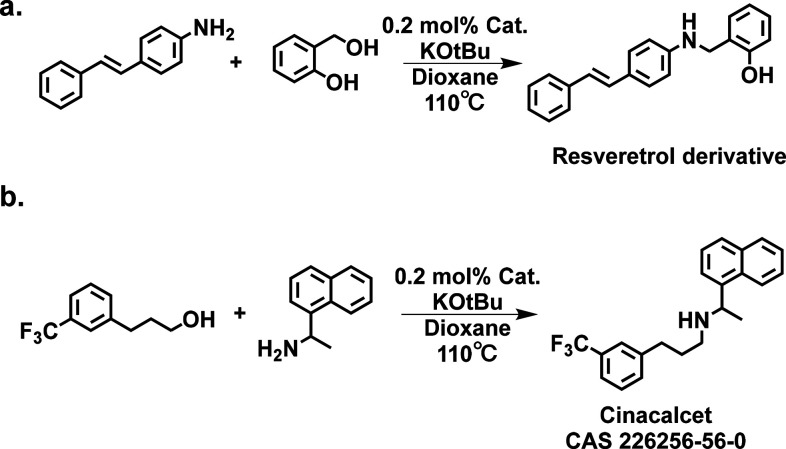
Synthesis of Pharmaceutical Model Compounds, (a) Resveratrol Derivative and (b) Cinacalcet, through N-Alkylation Catalyzed by MOF-Supported UiO-66–PPh_2_–Ir

Encouraged by the above results, the UiO-66–PPh_2_–Ir catalyst was used in the synthesis of pharmaceutical relevant molecules. Two pharmaceutical precursors, the resveratrol derivative and cinacalcet, were obtained with 95% conversion and 99% selectivity (Scheme 2 and Figure S14 of the Supporting Information). We further calculated the sizes of two medicinal precursors (Figure S15 of the Supporting Information), which are bigger than the pocket size of UiO-66 (around 7 Å). The high yield of two products is an indication of the surface-anchoring nature of the reactive complex. More importantly, high phase purity with no Ir residue was maintained in the mother liquor after centrifuging out the catalyst, as confirmed by inductively coupled plasma mass spectrometry (ICP–MS), which illustrates the potential of the catalyst for sustainable synthesis and medicinal chemistry.

### 3.3 Mechanistic Study

**Figure 2:**
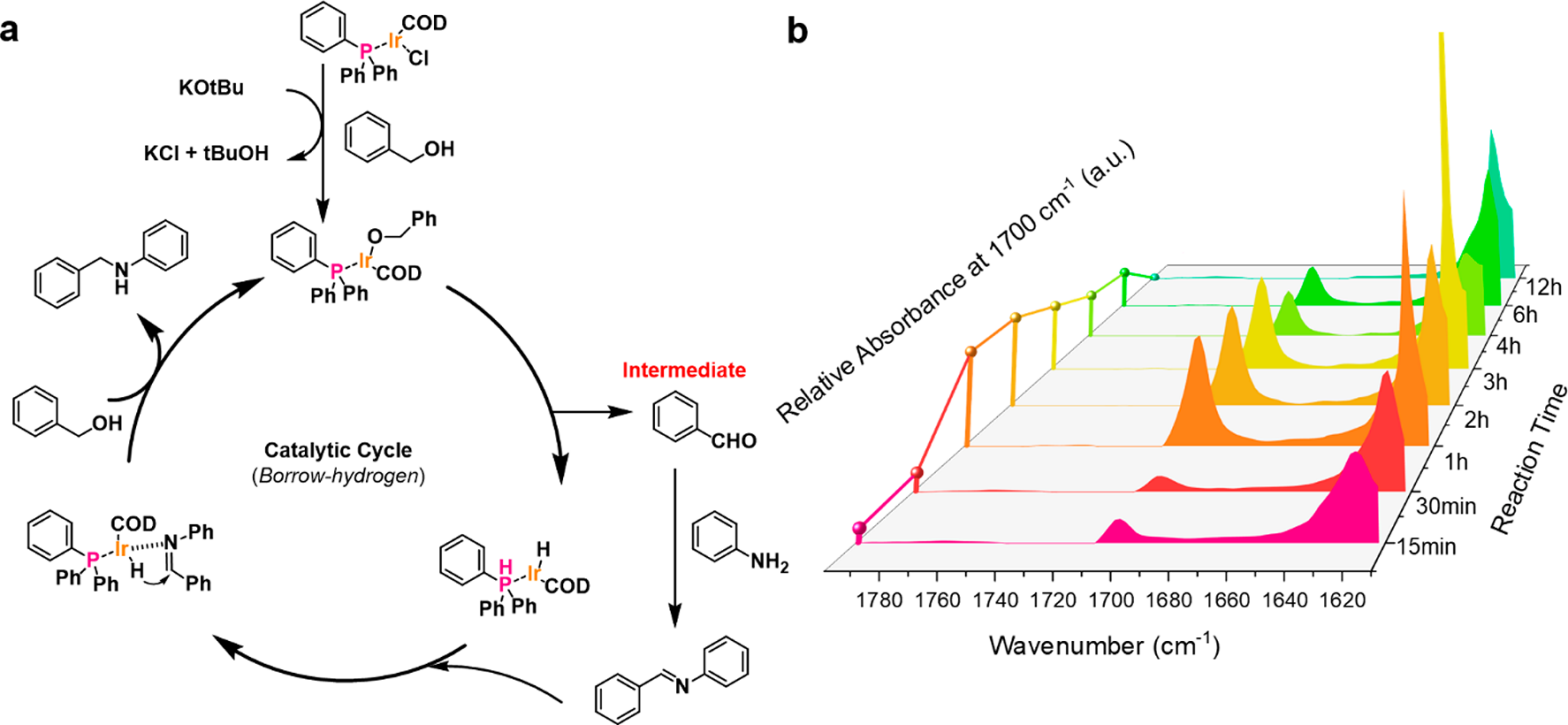
(a) Schematic presentation of the proposed mechanism through the borrowing hydrogen pathway. (b) Time-resolved IR spectrum of the N-alkylation reaction over 12 h and relative absorbance at 1700 cm^–1^.

The dihydrogen condensation reaction catalytic mechanism through a borrowing hydrogen pathway has been well-studied for homogeneous systems,^48,50−52^ in which the reaction intermediate could be monitored through *in situ* characterization methods. The borrowing hydrogen reaction pathway was experimentally confirmed by time-resolved IR, where the concentration of the aldehyde intermediate (C=O peak at 1700 cm^–1^ wavenumber) reaches a maximum at around 2 h and disappears during the course of the reaction. This temporal profile is consistent with the dehydrogenation, condensation, and rehydrogenation mechanism shown in Figure 2. These observations were further corroborated with the control experiment, where only alcohol oxidation was conducted. After 2 h of reaction with benzyl alcohol and KO*t*Bu in the presence of the UiO-66–PPh_2_–Ir catalyst, only 35% conversion to benzaldehyde was observed (entry 14 in Table S5 of the Supporting Information), even lower than the conversion of alcohol for the borrowing hydrogenation reaction, which showed that alcohol oxidation is the limiting reaction for the transformation. The low conversion may be ascribed to the accumulation of hydrogen gas, which pushes the chemical equilibrium backward. This enhancement of the reaction in the presence of amine is likely due to the timely removal of product (aldehyde and H_2_ gas) from the system pushing the reaction balance further. The reaction catalytic cycle was previously proposed for both homo- and heterogeneous Ir catalysts, in which Ir first binds the deprotonated alcohol through ligand exchange. The obtained complex undergoes the β-hydrogen elimination to achieve oxidation of alcohol to carbonyl, forming an Ir hydride intermediate. The obtained carbonyl compound undergoes condensation with amine to afford the imine product, which is rehydrogenated by Ir–H to produce substituted amine.^38^

## 4 Conclusion

In this report, we prepared Ir–phosphine-grafted UiO-66 MOFs for application in the N-alkylation of benzyl alcohol with aniline. The materials were characterized to demonstrate the incorporation of the Ir(I) sites on the surface of MOFs. UiO-66–PPh_2_–Ir has shown the highest catalytic activity and selectivity for this transformation. Kinetic studies reveal an induction period observed when using NHP complexes as a catalyst. Time-resolved IR and ultraviolet (UV) studies reveal that the N-alkylation reaction occurs via borrowing hydrogen from the alcohol substrate through the formation of Ir hydride and carbonyl intermediates. Finally, UiO-66–PPh_2_–Ir showed high stability after 4 consecutive runs in this transformation and a diverse substrate scope, including aliphatic and aromatic alcohol and amines. Subsequently, two pharmaceutical precursors were synthesized with up to 99% conversion and selectivity. This work demonstrates the advantages of MOF-supported complexes as heterogeneous catalysts that are amenable to molecular tuning and coordination control. The application in medicinal chemistry highlights their utility and potential to produce pharmaceuticals with a high purity.

## Supporting Information Available

Detailed descriptions of synthetic procedures and other experiments as well as analytical data (PDF)

## References

[ref1] Lin H.; Sun D. Recent Synthetic Developments and Applications of the Ullmann Reaction. A Review. Org. Prep. Proced. Int. 2013, 45, 341–394. 10.1080/00304948.2013.816208. PMC3819134 24223434

[ref2] Ruiz-Castillo P.; Buchwald S. L. Applications of Palladium-Catalyzed C–N Cross-Coupling Reactions. Chem. Rev. 2016, 116, 12564–12649. 10.1021/acs.chemrev.6b00512. 27689804 PMC5070552

[ref3] Müller T. E.; Hultzsch K. C.; Yus M.; Foubelo F.; Tada M. Hydroamination: Direct Addition of Amines to Alkenes and Alkynes. Chem. Rev. 2008, 108, 3795–3892. 10.1021/cr0306788. 18729420

[ref4] Irrgang T.; Kempe R. Transition-Metal-Catalyzed Reductive Amination Employing Hydrogen. Chem. Rev. 2020, 120, 9583–9674. 10.1021/acs.chemrev.0c00248. 32812752

[ref5] Bariwal J.; Van der Eycken E. C–N bond forming cross-coupling reactions: An overview. Chem. Soc. Rev. 2013, 42, 9283–9303. 10.1039/c3cs60228a. 24077333

[ref6] Joseph R.; Pallan P. S.; Sudalai A.; Ravindranathan T. Direct conversion of alcohols into the corresponding iodides. Tetrahedron Lett. 1995, 36, 609–612. 10.1016/0040-4039(94)02315-3.

[ref7] Guillena G.; Ramón D. J.; Yus M. Hydrogen Autotransfer in the N-Alkylation of Amines and Related Compounds using Alcohols and Amines as Electrophiles. Chem. Rev. 2010, 110, 1611–1641. 10.1021/cr9002159. 19928825

[ref8] Zhang Y.; Lim C.-S.; Sim D. S. B.; Pan H.-J.; Zhao Y. Catalytic Enantioselective Amination of Alcohols by the Use of Borrowing Hydrogen Methodology: Cooperative Catalysis by Iridium and a Chiral Phosphoric Acid. Angew. Chem., Int. Ed. 2014, 53, 1399–1403. 10.1002/anie.201307789. 24459057

[ref9] Rösler S.; Ertl M.; Irrgang T.; Kempe R. Cobalt-Catalyzed Alkylation of Aromatic Amines by Alcohols. Angew. Chem., Int. Ed. 2015, 54, 15046–15050. 10.1002/anie.201507955. 26474443

[ref10] Corma A.; Navas J.; Sabater M. J. Advances in One-Pot Synthesis through Borrowing Hydrogen Catalysis. Chem. Rev. 2018, 118, 1410–1459. 10.1021/acs.chemrev.7b00340. 29319294

[ref11] Celaje J. J. A.; Zhang X.; Zhang F.; Kam L.; Herron J. R.; Williams T. J. A Base and Solvent-Free Ruthenium-Catalyzed Alkylation of Amines. ACS Catal. 2017, 7, 1136–1142. 10.1021/acscatal.6b03088.

[ref12] Maji M.; Chakrabarti K.; Paul B.; Roy B. C.; Kundu S. Ruthenium(II)-NNN-Pincer-Complex-Catalyzed Reactions Between Various Alcohols and Amines for Sustainable C–N and C–C Bond Formation. Adv. Synth. Catal. 2018, 360, 722–729. 10.1002/adsc.201701117.

[ref13] Wong C. M.; Peterson M. B.; Pernik I.; McBurney R. T.; Messerle B. A. Highly Efficient Rh(I) Homo- and Heterogeneous Catalysts for C–N Couplings via Hydrogen Borrowing. Inorg. Chem. 2017, 56, 14682–14687. 10.1021/acs.inorgchem.7b02586. 29131601

[ref14] Mamidala R.; Mukundam V.; Dhanunjayarao K.; Venkatasubbaiah K. Cyclometalated palladium pre-catalyst for N-alkylation of amines using alcohols and regioselective alkylation of sulfanilamide using aryl alcohols. Tetrahedron 2017, 73, 2225–2233. 10.1016/j.tet.2017.03.001.

[ref15] Luo N.; Zhong Y.; Wen H.; Luo R. Cyclometalated Iridium Complex-Catalyzed N-Alkylation of Amines with Alcohols via Borrowing Hydrogen in Aqueous Media. ACS Omega 2020, 5, 27723–27732. 10.1021/acsomega.0c04192. 33134736 PMC7594325

[ref16] Reed-Berendt B. G.; Latham D. E.; Dambatta M. B.; Morrill L. C. Borrowing Hydrogen for Organic Synthesis. ACS Cent. Sci. 2021, 7, 570–585. 10.1021/acscentsci.1c00125. 34056087 PMC8155478

[ref17] Hao M.; Li Z. Efficient Visible Light Initiated One-Pot Syntheses of Secondary Amines from Nitro Aromatics and Benzyl Alcohols over Pd@NH_2_-UiO-66(Zr). Appl. Catal., B 2022, 305, 121031 10.1016/j.apcatb.2021.121031.

[ref18] Furukawa S.; Suzuki R.; Komatsu T. Selective Activation of Alcohols in the Presence of Reactive Amines over Intermetallic PdZn: Efficient Catalysis for Alcohol-Based N-Alkylation of Various Amines. ACS Catal. 2016, 6, 5946–5953. 10.1021/acscatal.6b01677.

[ref19] Jing W.; Shen H.; Qin R.; Wu Q.; Liu K.; Zheng N. Surface and Interface Coordination Chemistry Learned from Model Heterogeneous Metal Nanocatalysts: From Atomically Dispersed Catalysts to Atomically Precise Clusters. Chem. Rev. 2023, 123, 5948–6002. 10.1021/acs.chemrev.2c00569. 36574336

[ref20] Liu X.; Hermange P.; Ruiz J.; Astruc D. Pd/C as an Efficient and Reusable Catalyst for the Selective N-Alkylation of Amines with Alcohols. ChemCatChem 2016, 8, 1043–1045. 10.1002/cctc.201501346.

[ref21] Rojas-Buzo S.; Concepción P.; Corma A.; Moliner M.; Boronat M. In-Situ-Generated Active Hf-hydride in Zeolites for the Tandem N-Alkylation of Amines with Benzyl Alcohol. ACS Catal. 2021, 11, 8049–8061. 10.1021/acscatal.1c01739.

[ref22] Shimizu K.-i.; Imaiida N.; Kon K.; Hakim Siddiki S. M. A.; Satsuma A. Heterogeneous Ni Catalysts for N-Alkylation of Amines with Alcohols. ACS Catal. 2013, 3, 998–1005. 10.1021/cs4001267.

[ref23] Bavykina A.; Kolobov N.; Khan I. S.; Bau J. A.; Ramirez A.; Gascon J. Metal–Organic Frameworks in Heterogeneous Catalysis: Recent Progress, New Trends, and Future Perspectives. Chem. Rev. 2020, 120, 8468–8535. 10.1021/acs.chemrev.9b00685. 32223183

[ref24] Yuan S.; Zhang P.; Zhang L.; Garcia-Esparza A. T.; Sokaras D.; Qin J.-S.; Feng L.; Day G. S.; Chen W.; Drake H. F.; Elumalai P.; Madrahimov S. T.; Sun D.; Zhou H.-C. Exposed Equatorial Positions of Metal Centers via Sequential Ligand Elimination and Installation in MOFs. J. Am. Chem. Soc. 2018, 140, 10814–10819. 10.1021/jacs.8b04886. 30089362

[ref25] Chen W.; Cai P.; Elumalai P.; Zhang P.; Feng L.; Al-Rawashdeh M. m.; Madrahimov S. T.; Zhou H.-C. Site-Isolated Azobenzene-Containing Metal–Organic Framework for Cyclopalladated Catalyzed Suzuki–Miyuara Coupling in Flow. ACS Appl. Mater. Interfaces 2021, 13, 51849–51854. 10.1021/acsami.1c03607. 33914510

[ref26] Li H.; Xiong C.; Fei M.; Ma L.; Zhang H.; Yan X.; Tieu P.; Yuan Y.; Zhang Y.; Nyakuchena J.; Huang J.; Pan X.; Waegele M. M.; Jiang D.-e.; Wang D. Selective Formation of Acetic Acid and Methanol by Direct Methane Oxidation Using Rhodium Single-Atom Catalysts. J. Am. Chem. Soc. 2023, 145, 11415–11419. 10.1021/jacs.3c03113. 37172099

[ref27] Dhakshinamoorthy A.; Asiri A. M.; Garcia H. Metal–organic frameworks catalyzed C–C and C–heteroatom coupling reactions. Chem. Soc. Rev. 2015, 44, 1922–1947. 10.1039/C4CS00254G. 25608717

[ref28] Elumalai P.; Mamlouk H.; Yiming W.; Feng L.; Yuan S.; Zhou H.-C.; Madrahimov S. T. Recyclable and Reusable Heteroleptic Nickel Catalyst Immobilized on Metal–Organic Framework for Suzuki-Miyaura Coupling. ACS Appl. Mater. Interfaces 2018, 10, 41431–41438. 10.1021/acsami.8b16136. 30398346

[ref29] Mandal S.; Natarajan S.; Mani P.; Pankajakshan A. Post-Synthetic Modification of Metal–Organic Frameworks Toward Applications. Adv. Funct. Mater. 2021, 31, 2006291 10.1002/adfm.202006291.

[ref30] Drake T.; Ji P.; Lin W. Site Isolation in Metal–Organic Frameworks Enables Novel Transition Metal Catalysis. Acc. Chem. Res. 2018, 51, 2129–2138. 10.1021/acs.accounts.8b00297. 30129753

[ref31] Xu Y.; Mingos D. M. P.; Brown J. M. Crabtree’s catalyst revisited; Ligand effects on stability and durability. Chem. Commun. 2008, 199–201. 10.1039/B711979H. 18092086

[ref32] Beletskaya I. P.; Cheprakov A. V. The Heck Reaction as a Sharpening Stone of Palladium Catalysis. Chem. Rev. 2000, 100, 3009–3066. 10.1021/cr9903048. 11749313

[ref33] Guo H.; Fan Y. C.; Sun Z.; Wu Y.; Kwon O. Phosphine Organocatalysis. Chem. Rev. 2018, 118, 10049–10293. 10.1021/acs.chemrev.8b00081. 30260217 PMC6218176

[ref34] Fleming J. T.; Higham L. J. Primary phosphine chemistry. Coordin. Chem. Rev. 2015, 297–298, 127–145. 10.1016/j.ccr.2015.03.002.

[ref35] Peña-López M.; Neumann H.; Beller M. (Enantio)selective Hydrogen Autotransfer: Ruthenium-Catalyzed Synthesis of Oxazolidin-2-ones from Urea and Diols. Angew. Chem., Int. Ed. 2016, 55, 7826–7830. 10.1002/anie.201600698. 27072612

[ref36] Yang L. C.; Wang Y. N.; Zhang Y.; Zhao Y. Acid-Assisted Ru-Catalyzed Enantioselective Amination of 1,2-Diols through Borrowing Hydrogen. ACS Catal. 2017, 7, 93–97. 10.1021/acscatal.6b02959.

[ref37] Morris W.; Briley W. E.; Auyeung E.; Cabezas M. D.; Mirkin C. A. Nucleic Acid-Metal Organic Framework (MOF) Nanoparticle Conjugates. J. Am. Chem. Soc. 2014, 136, 7261–7264. 10.1021/ja503215w. 24818877

[ref38] Wong C. M.; McBurney R. T.; Binding S. C.; Peterson M. B.; Gonçales V. R.; Gooding J. J.; Messerle B. A. Iridium(iii) homo- and heterogeneous catalysed hydrogen borrowing C–N bond formation. Green Chem. 2017, 19, 3142–3151. 10.1039/C7GC01007A.

[ref39] Fliedel C.; Ghisolfi A.; Braunstein P. Functional Short-Bite Ligands: Synthesis, Coordination Chemistry, and Applications of N-Functionalized Bis(diaryl/dialkylphosphino)amine-type Ligands. Chem. Rev. 2016, 116, 9237–9304. 10.1021/acs.chemrev.6b00153. 27456550

[ref40] Zheng Z.; Yuan C.; Sun M.; Dong J.; Liu Y.; Cui Y. Construction of Monophosphine-Metal Complexes in Privileged Diphosphine-Based Covalent Organic Frameworks for Catalytic Asymmetric Hydrogenation. J. Am. Chem. Soc. 2023, 145, 6100–6111. 10.1021/jacs.2c11037. 36898039

[ref41] Yao W.; Duan Z.-C.; Zhang Y.; Sang X.; Xia X.-F.; Wang D. Iridium Supported on Phosphorus-Doped Porous Organic Polymers: Active and Recyclable Catalyst for Acceptorless Dehydrogenation and Borrowing Hydrogen Reaction. Adv. Synth. Catal. 2019, 361 (24), 5695–5703. 10.1002/adsc.201900929.

[ref42] Kramer S.; Bennedsen N. R.; Kegnæs S. Porous Organic Polymers Containing Active Metal Centers as Catalysts for Synthetic Organic Chemistry. ACS Catal. 2018, 8, 6961–6982. 10.1021/acscatal.8b01167.

[ref43] Bohigues B.; Rojas-Buzo S.; Moliner M.; Corma A. Coordinatively Unsaturated Hf-MOF-808 Prepared via Hydrothermal Synthesis as a Bifunctional Catalyst for the Tandem N-Alkylation of Amines with Benzyl Alcohol. ACS Sustainable Chem. Eng. 2021, 9, 15793–15806. 10.1021/acssuschemeng.1c04903. 35663357 PMC9153058

[ref44] Li J.; Yu M.; Duan Z.-C.; Zhu H.; Yao W.; Wang D. Porous cross-linked polymer copper and iridium catalyzed the synthesis of quinoxalines and functionalized ketones under solvent-free conditions. Mater. Chem. Front. 2021, 5, 7861–7872. 10.1039/D1QM00792K.

[ref45] Pintado-Sierra M.; Rasero-Almansa A. M.; Corma A.; Iglesias M.; Sánchez F. Bifunctional iridium-(2-aminoterephthalate)–Zr-MOF chemoselective catalyst for the synthesis of secondary amines by one-pot three-step cascade reaction. J. Catal. 2013, 299, 137–145. 10.1016/j.jcat.2012.12.004.

[ref46] Rasero-Almansa A. M.; Corma A.; Iglesias M.; Sánchez F. Design of a Bifunctional Ir–Zr Based Metal–Organic Framework Heterogeneous Catalyst for the N-Alkylation of Amines with Alcohols. ChemCatChem. 2014, 6, 1794–1800. 10.1002/cctc.201402101.

[ref47] Rasero-Almansa A. M.; Corma A.; Iglesias M.; Sánchez F. Post-functionalized iridium–Zr-MOF as a promising recyclable catalyst for the hydrogenation of aromatics. Green Chem. 2014, 16, 3522–3527. 10.1039/C4GC00581C.

[ref48] Podyacheva E.; Afanasyev O. I.; Vasilyev D. V.; Chusov D. Borrowing Hydrogen Amination Reactions: A Complex Analysis of Trends and Correlations of the Various Reaction Parameters. ACS Catal. 2022, 12, 7142–7198. 10.1021/acscatal.2c01133.

[ref49] Wang D.; Li Z. Coupling MOF-based photocatalysis with Pd catalysis over Pd@MIL-100(Fe) for efficient N-alkylation of amines with alcohols under visible light. J. Catal. 2016, 342, 151–157. 10.1016/j.jcat.2016.07.021.

[ref50] Zhao G.-M.; Liu H.-l.; Huang X.-r.; Zhang D.-d.; Yang X. Mechanistic study on the Cp*iridium-catalyzed N-alkylation of amines with alcohols. RSC Adv. 2015, 5, 22996–23008. 10.1039/C5RA02052B.

[ref51] Kallmeier F.; Fertig R.; Irrgang T.; Kempe R. Chromium-Catalyzed Alkylation of Amines by Alcohols. Angew. Chem., Int. Ed. 2020, 59, 11789–11793. 10.1002/anie.202001704. PMC7384194 32187785

[ref52] Vayer M.; Morcillo S. P.; Dupont J.; Gandon V.; Bour C. Iron-Catalyzed Reductive Ethylation of Imines with Ethanol. Angew. Chem., Int. Ed. 2018, 57, 3228–3232. 10.1002/anie.201800328. 29393563

