# Supplementary material for: N-Alkylation through the Borrowing Hydrogen Pathway Catalyzed by the Metal–Organic Framework-Supported Iridium–Monophosphine Complex
Source: ACS Appl Mater Interfaces. 2024 Apr 17;17(12):17775–82. doi: 10.1021/acsami.4c02143 (PMC11955943; doi:10.1021/acsami.4c02143)
Supplement: Supplementary file 1 — am4c02143_si_001.pdf [file am4c02143_si_001.pdf]

# Supporting Information

# N-alkylation Through Borrowing Hydrogen Pathway Catalyzed by Metal-Organic Framework Supported Iridium-Monophosphine Complex

Wenmiao Chen,[a,b] Muhammad Sohail,[a,d] Yempally Veeranna,[a] Yihao Yang,[b] Ashfaq A. Bengali[a], Hong-Cai Zhou,[b,*] and, Sherzod T. Madrahimov[a,*]

a Department of Science, Texas A&M University at Qatar, Education City, P.O. Box 23874, Doha, Qatar

*sherzod.madrahimov@qatar.tamu.edu*

b Department of Chemistry, Texas A&M University, College Station, Texas 77843-3255, United States

*zhou@chem.tamu.edu*

c School of Materials Science and Engineering, China University of Petroleum (East China)

Qingdao 266580, P. R. China.

d Department of Natural Sciences, Faculty of Science and Engineering, Manchester Metropolitan University, M15 6BH United Kingdom

## S1. General Information.

All reactions involving air and moisture sensitive compounds were carried out in a glovebox under argon atmosphere. All reagents and solvents were commercially available and used as supplied without further purification, unless otherwise noted. All glassware was oven-dried before use.

PXRD was carried out with a Bruker D8-Focus Bragg–Brentano X-ray Powder Diffractometer equipped with a Cu sealed tube (λ=1.54178 Å) at 40 kV and 40 mA. SCXRD was measured on a Bruker Venture CMOS diffractometer equipped with a Cu-Kα sealed-tube X-ray source (λ=1.5406 Å). ICP-MS data were collected with a Perkin Elmer NexION 300D ICP-MS.

All flash-column chromatography was carried out using silica gel (MP Silitech 60-200 mesh, MP Biomedicals, LLC, Santa Ana, CA, USA) under a positive pressure of air, unless otherwise noted. Analytical thin layer chromatography (TLC) was performed using glass-backed silica gel 60 F254 plates (Merck EMD-571507, Whitehouse Station, NJ, USA) or aluminum oxide on plastic sheets (J.T. Baker Chemical Co, Phillipsburg, New Jersey). Visualization of TLC results was achieved by observation under UV light (254 nm).

Field-emission SEM images were collected on the FEI Quanta 600 field-emission SEM at 20 KV. Source: Field emission gun assembly with Schottky emitter source. Beam Current: >100 nA. The high resolution XPS measurements were performed with a Perkin Elmer PHI system. The sample was placed into the XPS chamber with a base pressure of <1.0 × 10−9 Torr. Al Kα (1486.6 eV) X-ray source at a chamber was used to excite photoelectrons. The spectra were recorded by using a 16-channel detector with a hemispherical analyzer. Micromeritics Belsorp-max analyzer was applied to measure the Brunauer Emmett Teller (BET) surface area and pore size distribution (PSD). The XAFS spectra at Ir-$L_3$ edge were acquired at 4B9A station in Beijing Synchrotron Radiation Facility (BSRF, operated at 2.5 GeV with a maximum current of 250 mA). The raw EXAFS data acquired were then background-subtracted, normalized, and Fourier-transformed by using the ATHENA program in the IFEFFIT software package. Least-squares curve parameter fitting was performed using the ARTEMIS module of IFEFFIT software packages. The Ir-$L_3$ edge XANES data were recorded in a fluorescence mode. Ir foil and $IrO_2$ were used as references.

$^{1}H$, $^{13}C$ and $^{31}P$ NMR spectra were recorded on a Bruker Avance 400 spectrometer and referenced to the residual solvent peak. $^{1}H$ NMR data are reported as follows: chemical shift (multiplicity (bs = broad singlet, s = singlet, d = doublet, t = triplet, q = quartet, p = pentet and m = multiplet), coupling constant and integration). $^{1}H$, $^{13}C$ and $^{31}P$ NMR chemical shifts are reported in ppm downfield from tetramethylsilane (TMS, δ scale) using the residual solvent resonances as internal standards.

$^{31}P$ NMR details:

To get the accurate quantitative ratio of the peak corresponding to the MOF to that of external standard in a capillary tube, $^{31}P$ NMR was measured at 400MHz, and 1024 scans, relaxation delay 4 s, an acquisition time of 0.498 sec.

X-ray absorption (XAS) data of the Ir $L_3$-edge was measured at station 4B9A of the Beijing Synchrotron Radiation Facility (BSRF, working at 2.5 GeV, with a maximum current of 250 mA). The X-ray beam was monochromatized by a Si (111) monochromator and detuned by 50% to reduce the contribution of higher-order harmonics below the level of noise. Zn foil standard was used as a reference for energy calibration and was measured simultaneously with experimental samples. Sample UiO-66-$PPh_2$-Ir was ground and measured in transmission mode using a Lytle detector. The obtained XAFS data were processed in Athena (version 0.9.26) and Fourier transform fitting was carried out using software Artemis (version 0.9.26) in the IFEFFIT package in R space, with a k-weight of 3.

All gas chromatography (GC) analyses at Texas A&M University at Qatar were conducted on a PerkinElmer Inc. - Clarus 500 GC Mass Spectrometer equipped with FID Elite-1 capillary column (30 m × 0.25 m × 0.25 µm film thickness). The GC/MS data were acquired using an Agilent 7890 GC/MSD Gas Chromatograph with 5975 Triple Axis MSD Detector and with a HP-5ms capillary column (30 m × 0.25 mm; film thickness 0.25 µm).

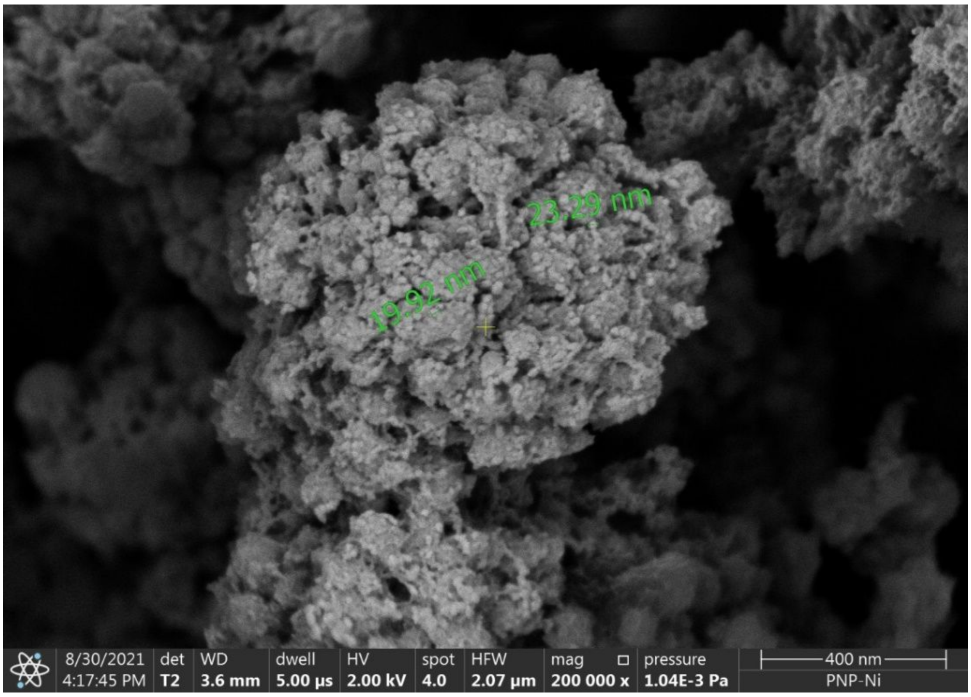


**Figure S1** SEM picture of UiO66 (nano), with particle sizes measured around 20nm (shown in green).

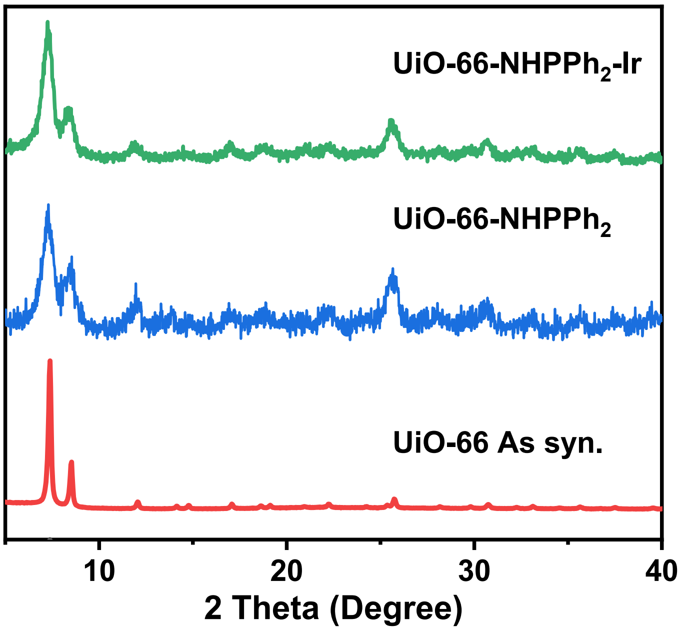


**Figure S2.** PXRD pattern of **UiO-66, UiO-66-NHPPh$_2$** and **UiO-66-NHPPh$_2$-Ir.**

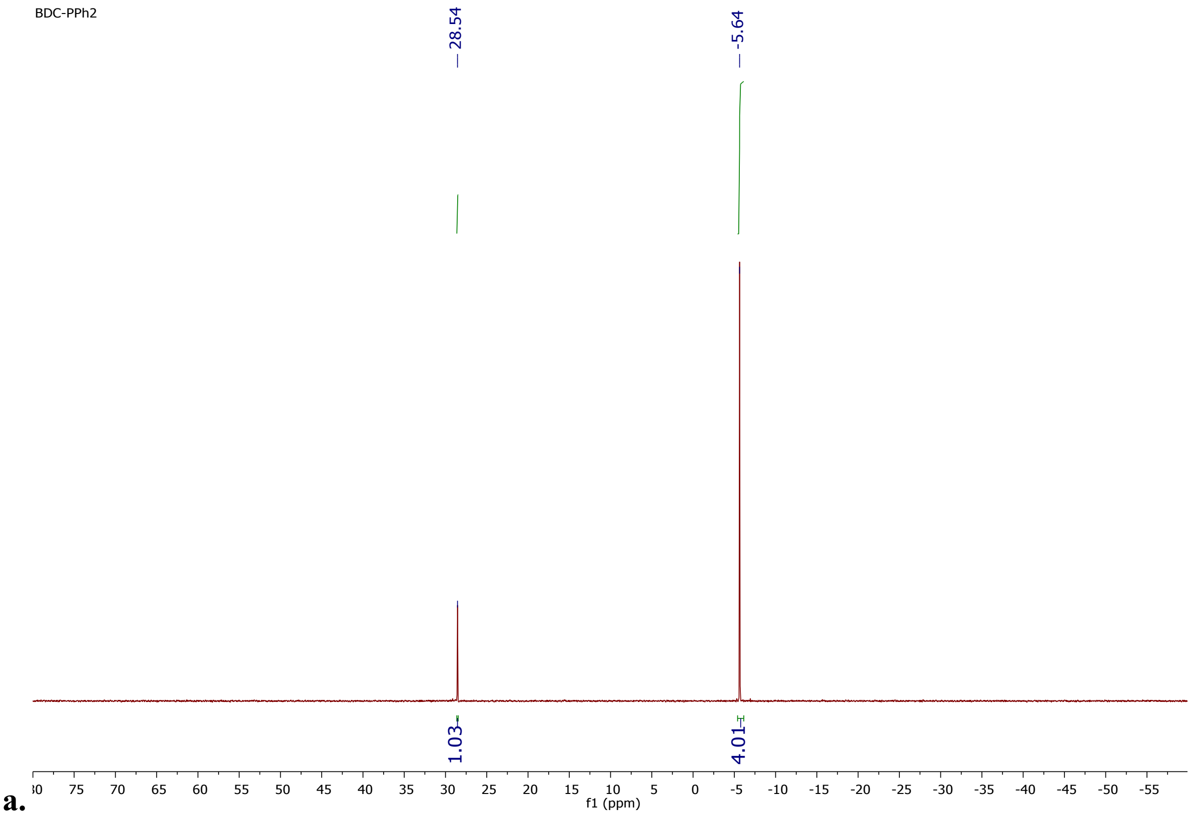


a.

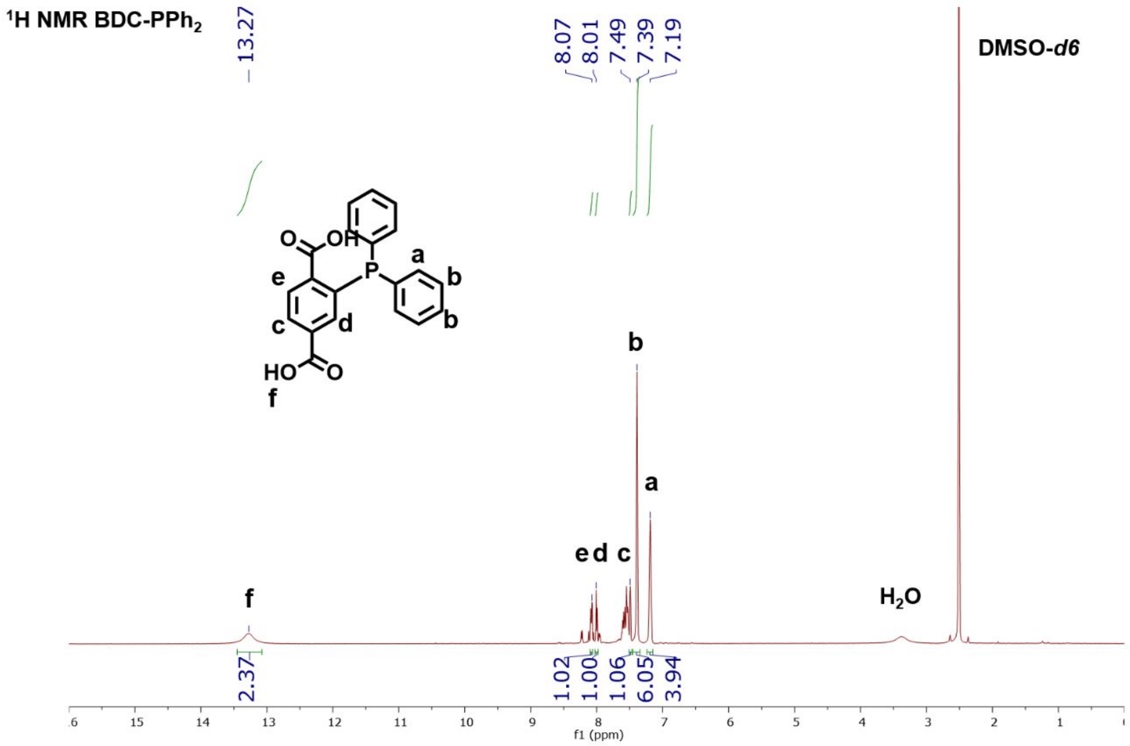


b.

**Figure S3.** a. $^{31}$P and b. $^{1}$H NMR for the **BDC-PPh$_2$** acid ligand in DMSO-*d6* (partially oxidized).

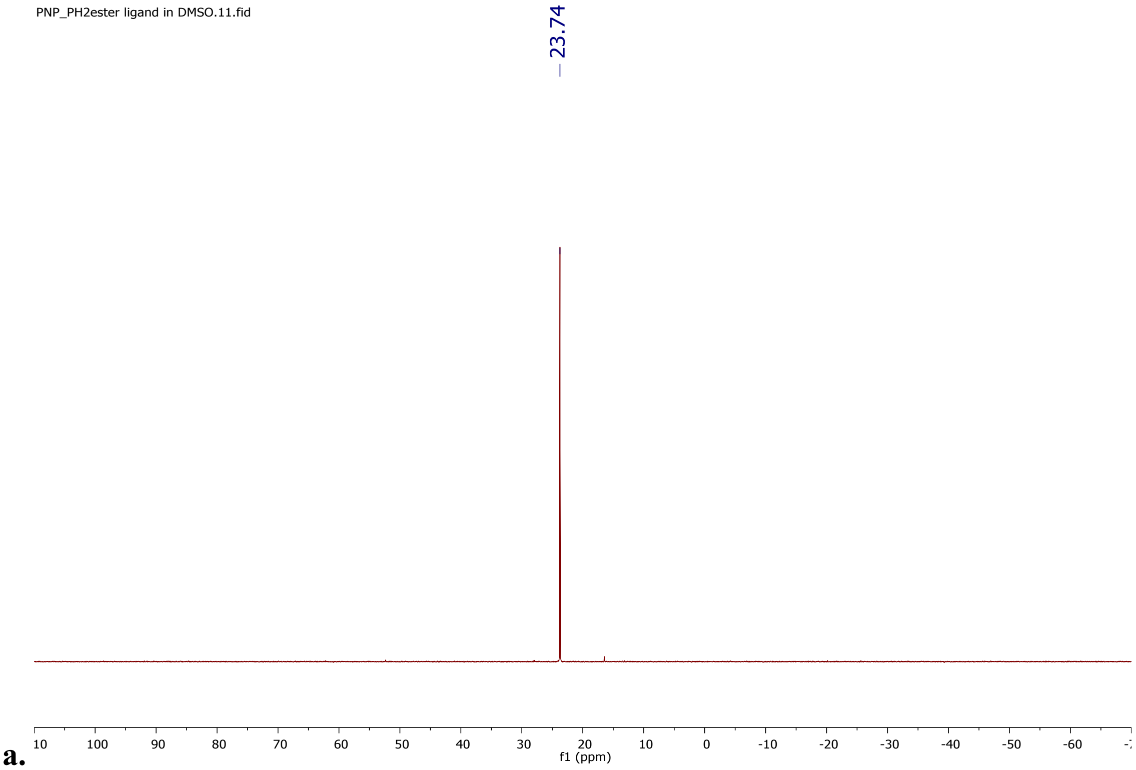


a.

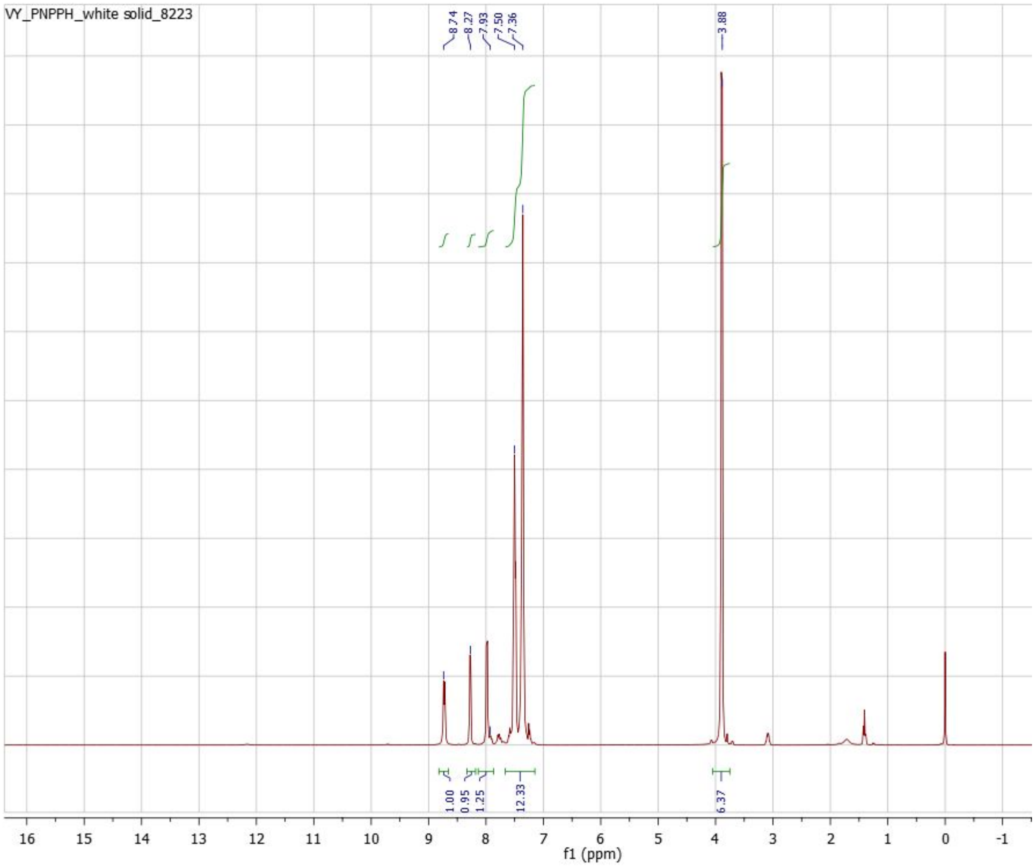


**b.**

**Figure S4.** a. $^{31}$P and b. $^{1}$H NMR of **BDC-NHPPh$_2$** ester ligand in DMSO-*d6*.

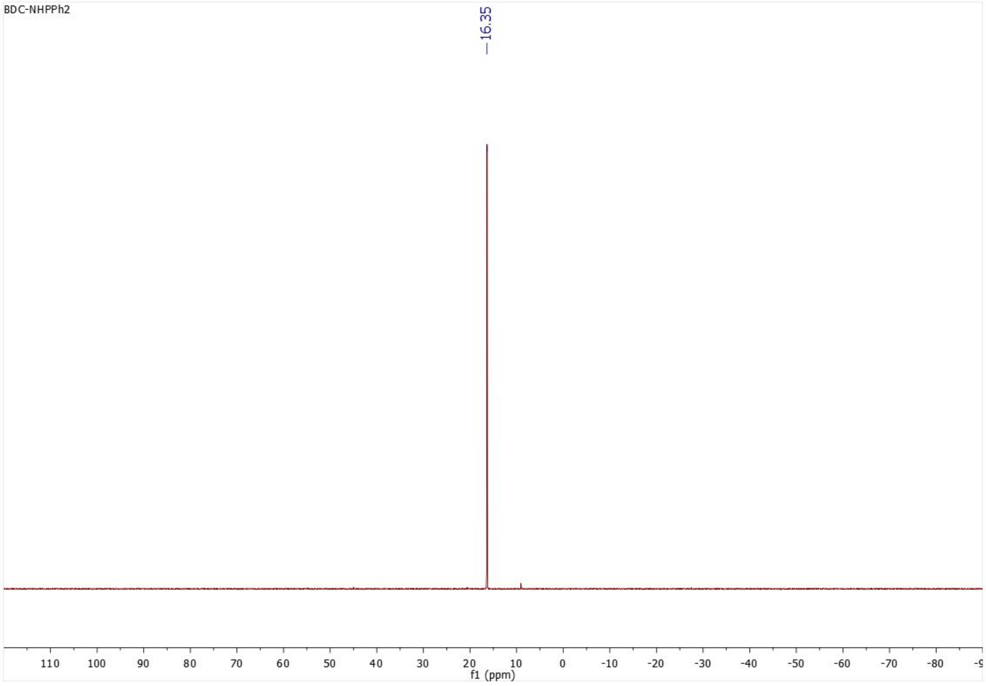


**a.**

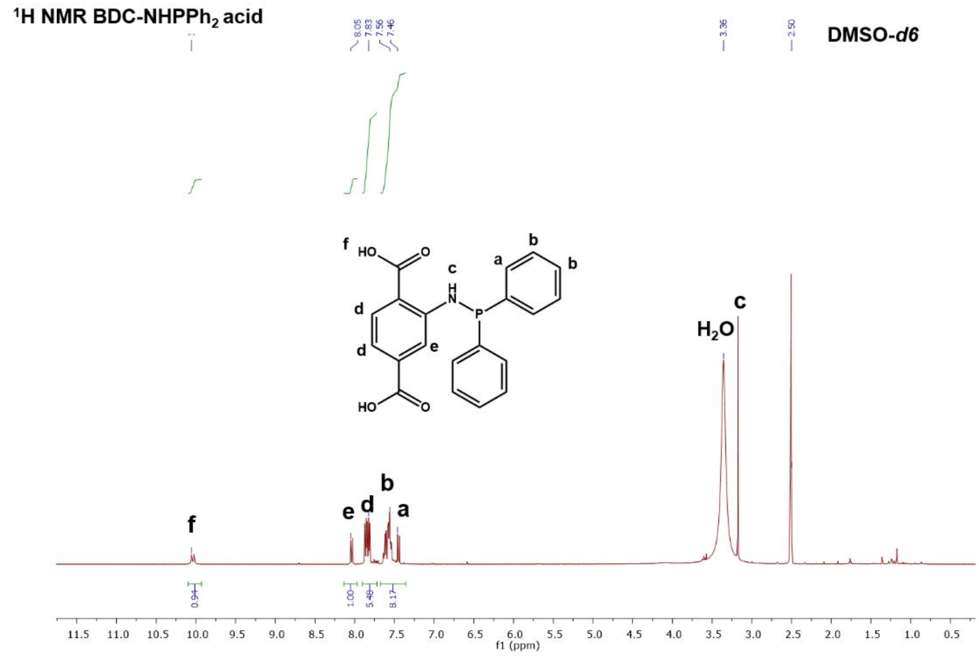


**b.**

**Figure S5.** a. $^{31}$P and b. $^{1}$H NMR of **BDC-NHPPh$_2$** acid ligand in DMSO-*d6*.

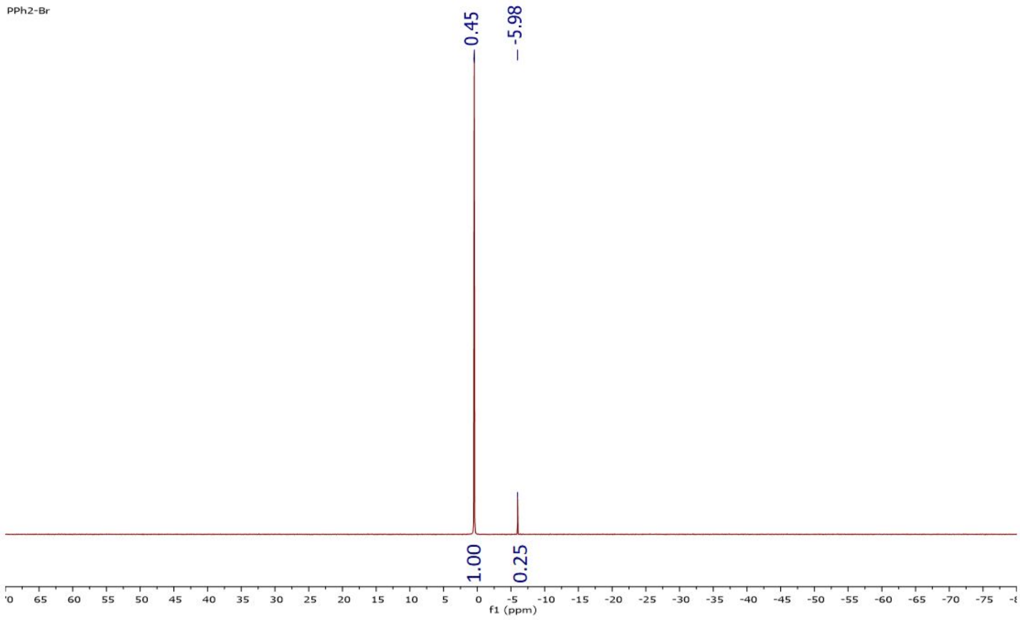


**a.**

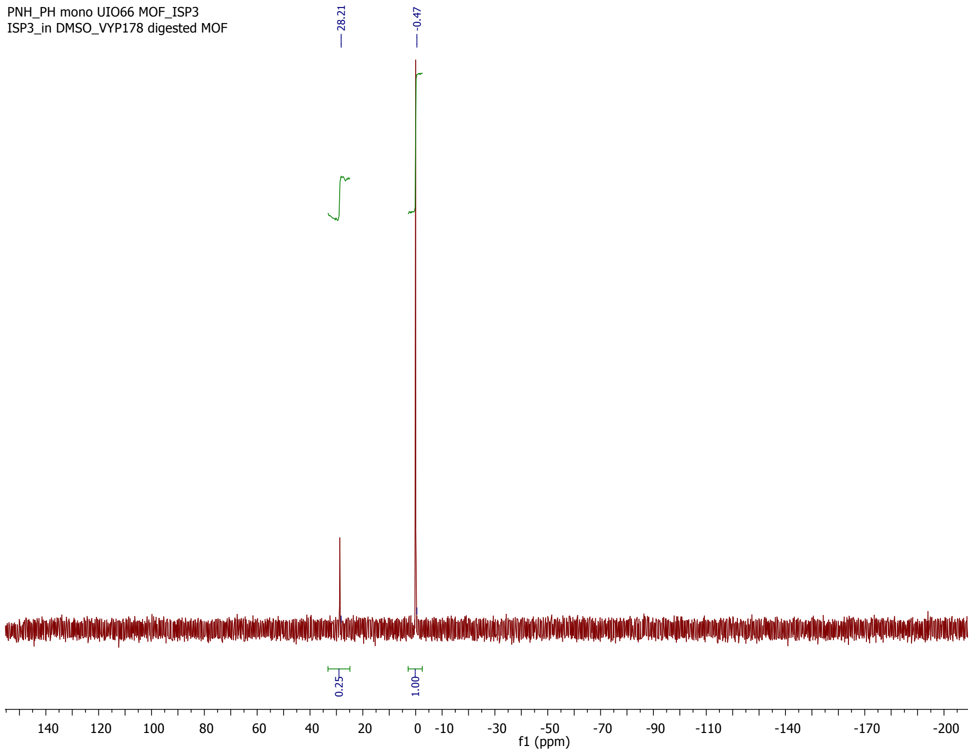


**b.**

**Figure S6.** $^{31}P$ for the **a. UiO-66-PPh$_2$** (digested under $N_2$ to prevent oxidation) **and b. UiO-66-NHPPh$_2$** digested in $D_2SO_4$/DMSO-*d6* with $H_3PO_4/D_2O$ external standard added in a capillary tube. $PPh_2$: shift -5.98 ppm, integration 0.25, Formula: UiO-66-$(PPh_2)_{1.5}$

$NHPPh_2$: shift 28.2 ppm, integration 0.25, Formula: UiO-66-$(NHPPh_2)_{1.5}$

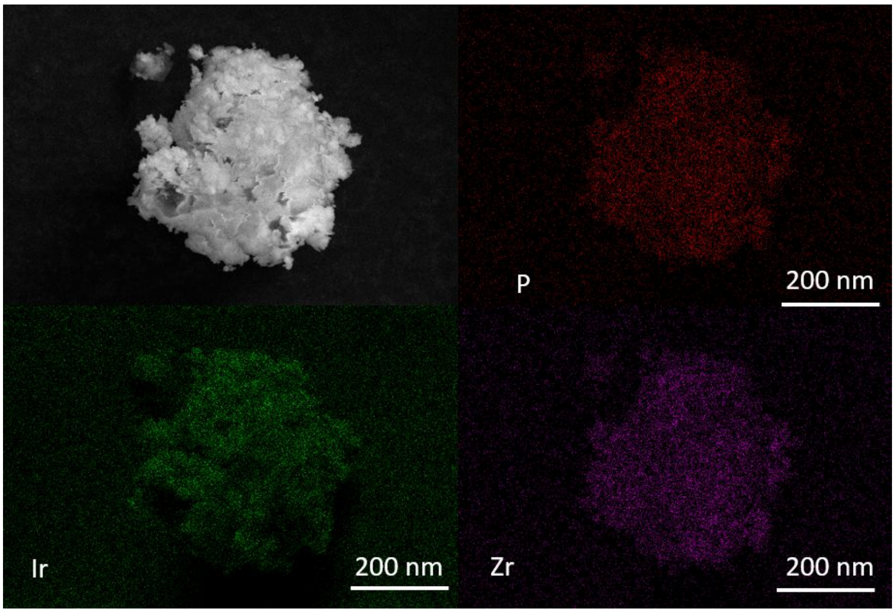


**Figure S7** EDS elemental mapping image of Zr, Ir and P of **UiO66-PPh$_2$-Ir**

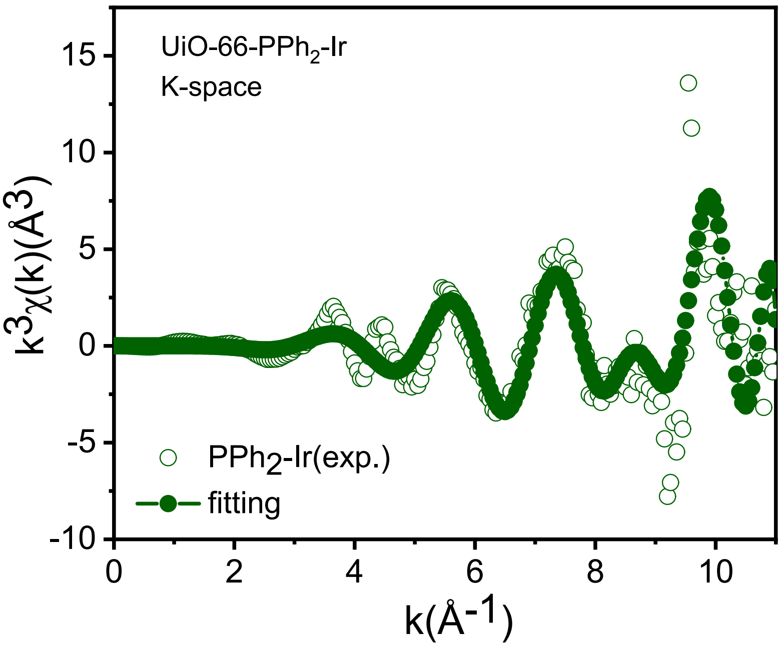


**Figure S8.** EXAFS data (circles) and best fits (solid lines) of **UiO-66-$PPh_2$-Ir** in k-space.

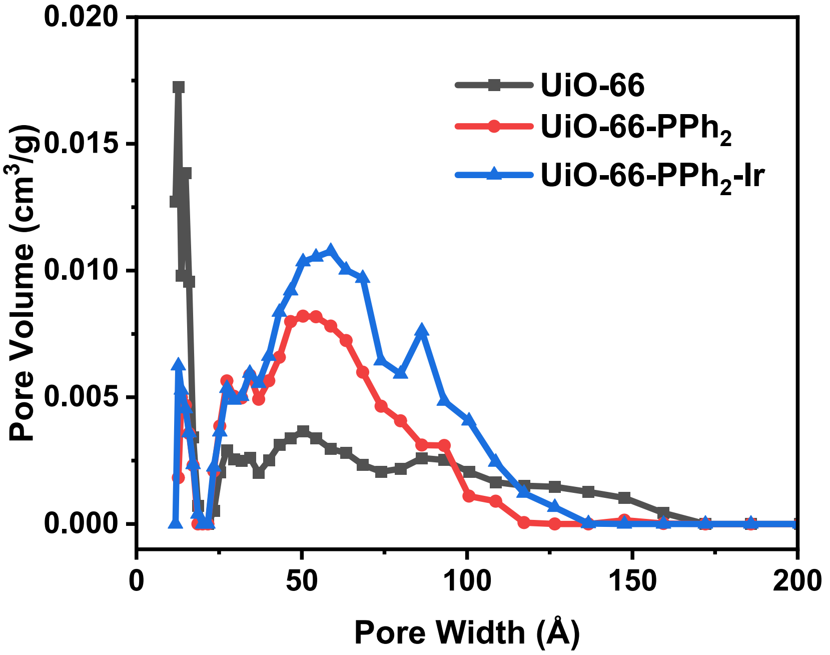


**Figure S9.** Pore size distribution analysis of **UiO-66, UiO-66-$PPh_2$** and **UiO-66-$PPh_2$-Ir**

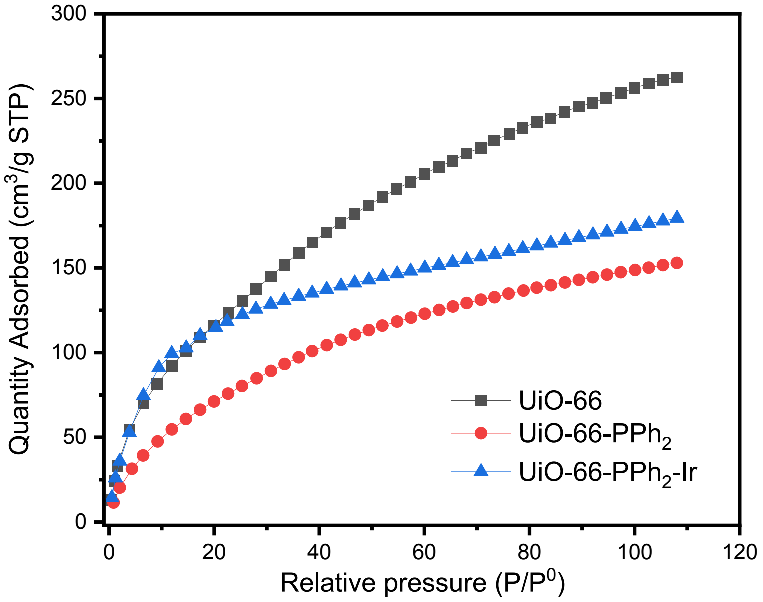


**Figure S10.** $H_2$ adsorption of **UiO-66, UiO-66-PPh$_2$** and **UiO-66-PPh$_2$-Ir.**

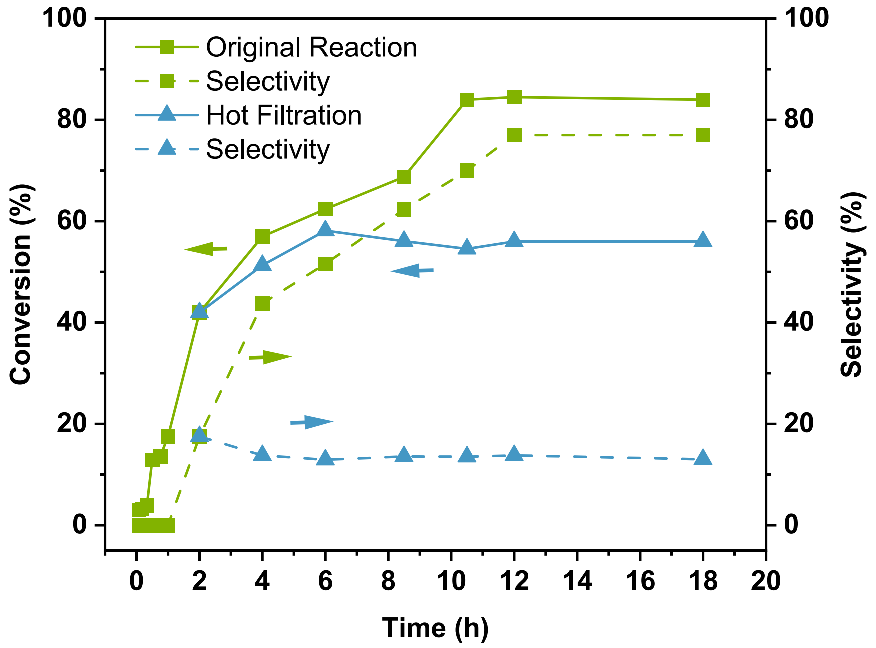


**Figure S11**. Yield and selectivity of the N-alkylation with and without hot filtration experiment.

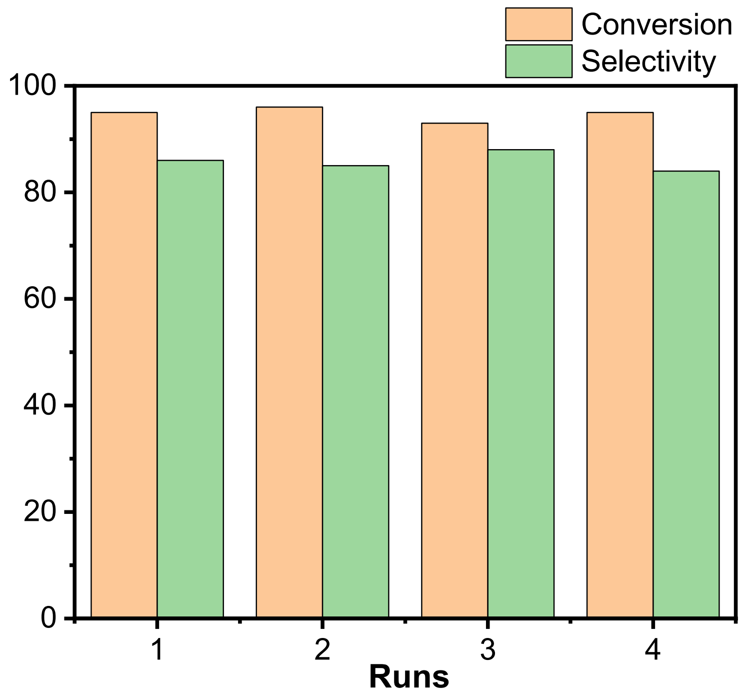


**Figure S12**. Recycle experiment of N-alkylation catalyzed by **UiO-66-$PPh_2$-Ir.**

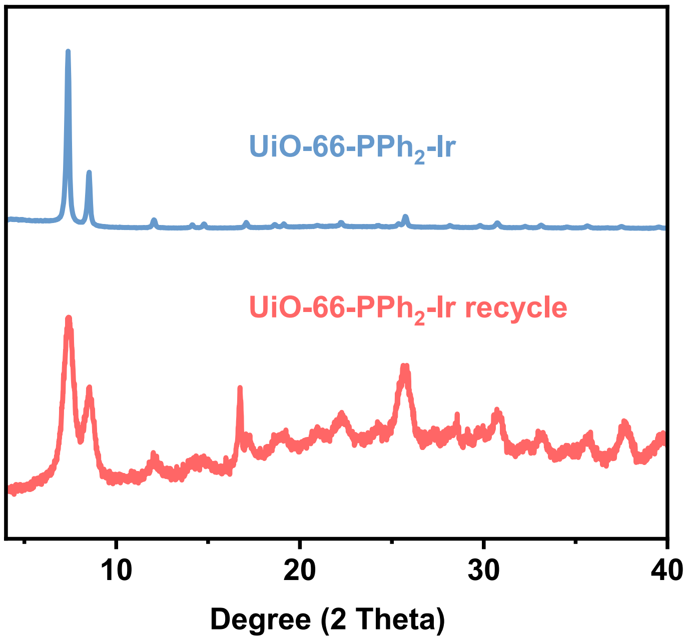


**Figure S13**. PXRD of **UiO-66-$PPh_2$-Ir** before and after 4 catalytic runs.

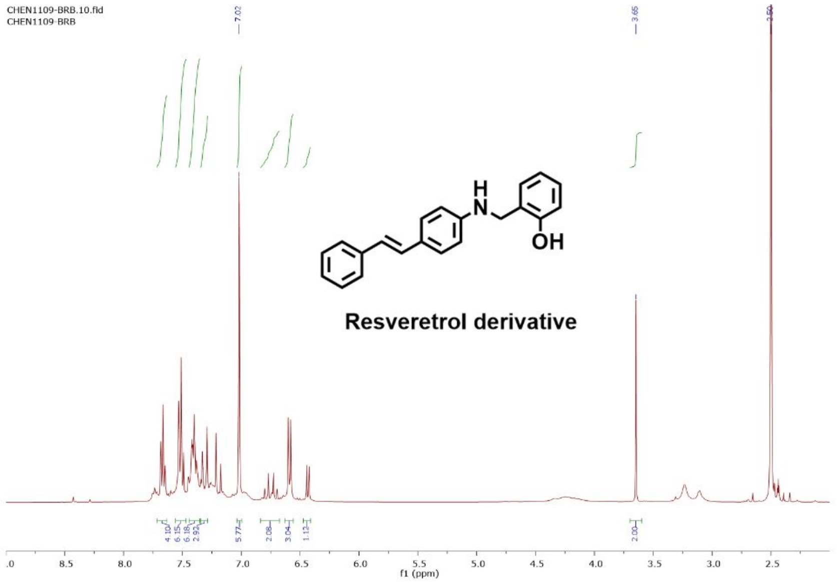


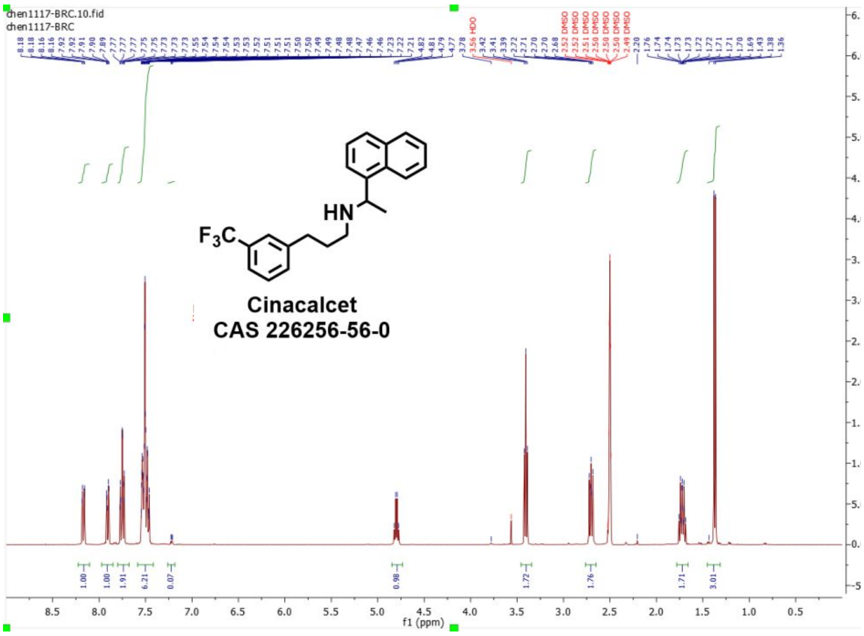


**Figure S14.** $^{1}$H-NMR of the medicine precursor a. resveratrol derivative and b. Cinacalcet.

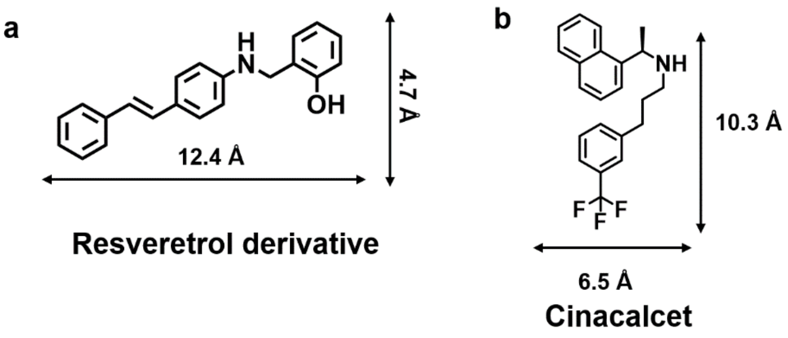


**Figure S15.** Schematic presentation of the sizes of a. Resveratrol derivative and b. Cinacalcet.

**Table S1**. SCXRD data and structure refinements of BDC-NHPPh$_2$ ester.

| Name | BDC-NHPPh$_2$ ester |
|---|---|
| **CCDC** | 2267911 |
| **Empirical formula** | $C_{22}H_{21}N_1O_4P_1$ |
| **Formula weight** | 394.39 |
| **Temperature/K** | 110 |
| **Crystal system** | triclinic |
| **Space group** | *P*-1 |
| **a/Å** | 8.577(2) |
| **b/Å** | 11.256(3) |
| **c/Å** | 11.906(3) |
| **α/°** | 66.454(5) |
| **β/°** | 73.037(6) |
| **γ/°** | 73.364(5) |
| **Volume/Å$^3$** | 988.8(4) |
| **Z** | 2 |
| **$\rho_{calc}$g/cm$^3$** | 1.325 |
| **μ/mm$^{-1}$** | 1.469 |
| **F(000)** | 415.949 |
| **Radiation** | Cu Kα (λ = 1.54184) |
| **2Θ range for data collection/°** | 9.74 to 144.34 |
| **Index ranges** | -9 ≤ h ≤ 10 -12 ≤ k ≤ 13, 0 ≤ l ≤ 14 |
| **Reflections collected** | 3790 |
| **Independent reflections** | 3790 [$R_{sigma}$ = 0.0762] |
| **Data/restraints/parameters** | 3860/0/337 |
| **Goodness-of-fit on F$^2$** | 1.053 |
| **Final R indexes [I>=2σ (I)]** | $R_1$ = 0.545, $wR_2$ = 0.1403 |
| **Final R indexes [all data]** | $R_1$ = 0.565, $wR_2$ = 0.1436 |
| **Largest diff. peak/hole / e Å$^{-3}$** | 0.4197/-0.6091 |

$R_1 = \Sigma||F_o| - |F_c||/\Sigma|F_o|$. $wR_2 = |\Sigma w(|F_o|^2 - |F_c|^2)|/\Sigma|w(F_o^2)^2|^{1/2}$

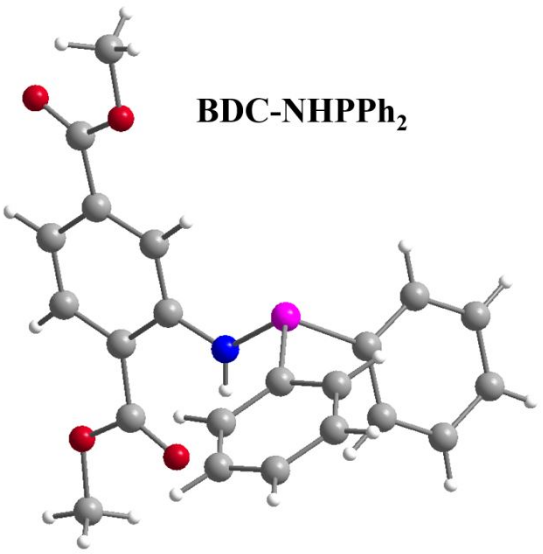

**Table S2**. Calculation of molecular weight of **UiO-66-PPh$_2$** and **NHPPh$_2$** from the $^{31}$P NMR

| | mass dissolved in acid (mg) | integration MOF | integration standard | C standard (M) | volume (mL) | concentration MOF (M) | millimoles MOF | MW MOF |
|---|---|---|---|---|---|---|---|---|
| **UiO-66-PPh$_2$** | 2.5 | 0.21 | 1.00 | 0.0158 | 0.8 | 0.0033 | 0.0027 | 1900 |
| **UiO-66-NHPPh$_2$** | 3.1 | 1.12 | 2.88 | 0.0137 | 0.5 | 0.0053 | 0.0026 | 2300 |

**Table S3.** Ir to Zr atomicratio from the ICP-MS.

| Sample Name | Ir Ratio | Zr Ratio | Result formula |
|---|---|---|---|
| **UiO-66-PPh$_2$-Ir** | 0.157 | 1 | UiO-66-(PPh$_2$)$_{1.5}$-Ir$_{1.0}$ |
| **UiO-66-NHPPh$_2$-Ir** | 0.164 | 1 | UiO-66-(NHPPh$_2$)$_{1.5}$-Ir$_{1.1}$ |
| **UiO-66-PPh$_2$-Ir recycled** | 0.153 | 1 | UiO-66-(PPh$_2$)$_{1.5}$-Ir$_{1.0}$ |

**Table S4**. Summary of EXAFS fitting parameters for **UiO-66-PPh$_2$-Ir**.

| **Sample** | **UiO-66-PPh$_2$-Ir** |
|---|---|
| **Fitting range** | k 3-12.331 Å$^{-1}$ R 1-3 Å |
| **Independent points** | 11.6 |
| **Variables** | 8 |
| **Reduced $\chi$2** | 571.4 |
| **R-factor** | 0.02 |
| **$S_0^2$** | 0.17 |
| **$\Delta E_0$(eV)** | 12.26383+/-2.41741 |
| **ΔR(C-Ir) (Å)** | 0.00799+/-0.03112 |
| **$\sigma^2$ (C-Ir) (Å$^2$)** | -0.00123+/-0.00399 |
| **ΔR(Cl-Ir) (Å)** | 0.00187+/-0.00420 |
| **$\sigma^2$ (Cl-Ir) (Å$^2$)** | -0.01748+/-0.00375 |
| **ΔR(P-Ir) (Å)** | 0.00466+/-0.00445 |
| **$\sigma^2$ (P-Ir) (Å$^2$)** | 0.01839+/-0.00407 |

**Table S5**. Screening of **UiO-66-$PPh_2$-Ir** Catalyst and condition optimization for N-alkylation reaction

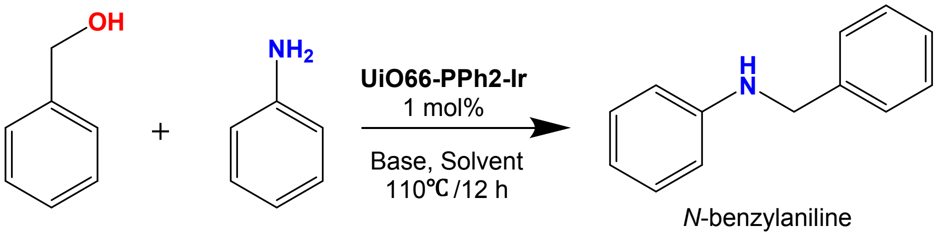


| entry | cat. | solvent | base | conversion[b]/selectivity |
|---|---|---|---|---|
| 1 | UiO66-$PPh_2$-Ir | toluene | KOtBu | 84(77) |
| 2 | UiO66-$PPh_2$-Ir | toluene | $K_2CO_3$ | 25(2) |
| 3 | UiO66-$PPh_2$-Ir | toluene | NaOtBu | 62(1) |
| 4 | UiO66-$PPh_2$-Ir | dioxane | KOtBu | 95(86) |
| 5[c] | UiO66-NHPipr-Ir | dioxane | KOtBu | 34(85) |
| 6 | UiO66-$PPh_2$-Ir | dioxane | KOtBu | 5(18) |
| 7 | UiO66 + Ir | dioxane | KOtBu | NA |
| 8 | UiO66-$PPh_2$ | dioxane | KOtBu | NA |
| 9[d] | Ir + $PPh_2$ | toluene | KOtBu | 94(23) |
| 10[e] | UiO66-$PPh_2$-Rh | dioxane | KOtBu | 78(90) |
| 11 | UiO66-Hf-$PPh_2$-Ir | dioxane | KOtBu | 95(90) |
| 12[f] | UiO66-NH$PPh_2$-Ir | dioxane | KOtBu | 65(90) |
| 13[g] | UiO66-$PPh_2$-Ir | dioxane | KOtBu | 90(88) |
| 14[h] | UiO66-$PPh_2$-Ir | dioxane | KOtBu | 35(0) |

[a]Reaction conditions: aniline (0.2 mmol), benzyl alcohol (0.3 mmol), solvent (2 mL), base (0.4 mmol), time (12 h), and MOF (1 mol %). [b]Conversion and selectivity were determined by GC-FID with mesitylene as internal standard. [c]Reaction under 60 ℃. [d]Organic linker and metal salt (1:1 molar ratio) was added instead. [e]Catalyst was prepared with Rh(DME)Cl precursor. [f]Catalyst was prepared with NH$PPh_2$ precursor. [g]Catalyst was prepared with NHPipr precursor [g]Control experiment with 0.2 mmol base, 2 mmol aniline and 3 mmol benzyl alcohol (0.1 equiv.). [h]Control experiment without aniline, only benzyl alcohol, base and catalyst were added.

## S3. Product Characterization.

Products Characterization for **UiO-66-$PPh_2$-Ir** Catalyzed N-alkylation reaction[2-4]

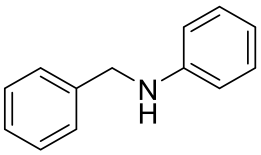


***N*-benzylaniline .** $^1$H NMR (500 MHz, $CDCl_3$): δ = 7.44-7.38 (m, 4H), 7.35-7.32 (m, 1H), 7.26 - 7.22 (m, 2H), 6.78 (m, 1H), 6.69 (d, 2H), 4.37(s, 2H), 4.12(s, 1H). $^{13}$C NMR(125 MHz, $CDCl_3$): δ = 148.14, 139.46, 129.34, 128.70, 127.59, 127.30, 117.69, 112.97, 48.41.

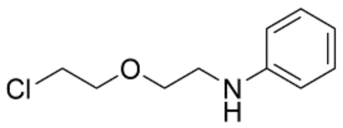


***N*-(2-chloroethoxyl)ethyl-aniline (1a).** $^1$H NMR (500 MHz, $CDCl_3$): δ = 9.63 (s, 1H), 7.11 (t, 2H), 6.67-6.56 (m, 3H), 3.84 (t, 2H), 3.67 (m, 4H), 3.44 (t, 2H). MW: 199.18.

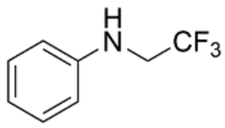


***N*-(4-trifluoroethyl)aniline (1b).** $^1$H NMR (500 MHz, $CDCl_3$): δ = 7.31-7.28 (d, 2H), 7.21-7.17 (m, 4H), 6.73 (m, 1H), 6.65 (d, 2H), 4.25 (s, 2H), 4.09 (s, 1H), 2.35 (s, 3H). MW: 175.15.

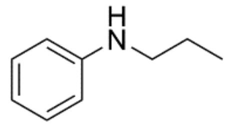


***N*-propyl-aniline (1c).** $^1$H NMR (500 MHz, $CDCl_3$): δ = 7.21-7.14 (m, 2H), 6.73-6.49 (m, 3H), 6.73 (m, 1H), 3.55 (s, 1H), 3.09 (t, 2H), 1.82-1.49 (m, 2H), 1.01 (t, 3H). MW: 135.16.

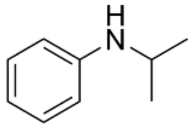


***N*-isopropylaniline (1d).** $^1$H NMR (500 MHz, $CDCl_3$): δ = 7.10-7.05 (m, 2H), 6.62-6.49 (m, 3H), 3.55 (m, 1H), 3.28 (s, 1H), 1.12 (d, 6H). MW: 135.21.

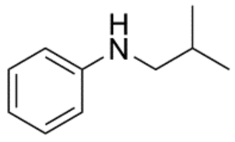


***N*-(2-methylpropyl)benzenamine (1e).** $^1$H NMR (500 MHz, $CDCl_3$): δ = 7.21-7.17 (m, 2H), 6.74-6.68 (m, 3H), 2.98 (d, 2H), 3.28 (s, 1H), 1.91 (m, 1H), 0.99 (d, 6H). MW: 149.25.

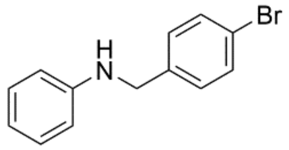

***N*-(4-bromobenzyl)aniline (1f).** $^{1}$H NMR (500 MHz, $CDCl_3$): δ = 7.51 (d, 2H), 7.29 (d, 2H), 7.23 (m, 2H), 6.79 (t, 1H), 6.66 (d, 2H), 4.33 (s, 2H). MW: 261.11.

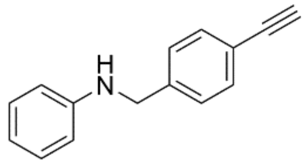

***N*-(4-ethynyl-phenyl)benzenamine (1g).** $^{1}$H NMR (500 MHz, $CDCl_3$): δ = 7.51 (s, 1H), 7.46-7.38 (m, 4H), 7.11 (t, 2H), 6.83-6.65 (m, 3H), 4.32 (s, 2H), 3.01 (s, 1H). MW: 207.27.

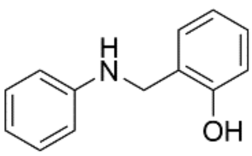

**2-[(phenylamino)methyl]-phenol (1h).** $^{1}$H NMR (500 MHz, $CDCl_3$): δ = 8.31 (s, 1H), 7.21-7.14 (m, 3H), 7.08 (d, 1H), 6.84-6.78 (m, 3H), 6.75 (d, 2H), 4.32 (s, 2H), 3.90 (s, 1H). MW: 199.25.

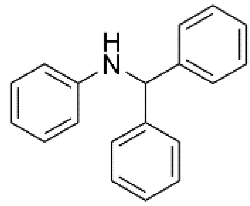

***N*-(Diphenylmethyl)aniline (1i).** $^{1}$H NMR (500 MHz, $CDCl_3$): δ = 7.34-7.23 (m, 10H), 7.13-7.10 (m, 2H), 6.74-6.69 (m, 1H), 6.29-6.56 (m, 2H), 5.60 (s, 1H). MW: 259.38.

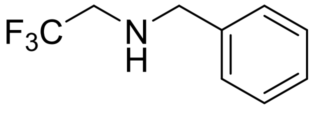

***N*-benzyl-trifluoro ethyl-aniline (1j).** $^{1}$H NMR (500 MHz, $CDCl_3$): δ = 7.85-7.83 (m, 2H), 7.34-7.31 (d, 2H), 7.25-7.21 (m, 5H), 4.50 (s, 1H). MW: 190.1.

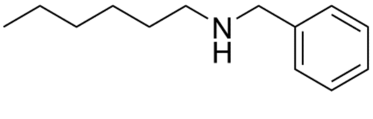

***N*-hexylaniline (1k).** $^{1}$H NMR (500 MHz, $CDCl_3$): δ = 8.27 (s, 1H), 7.74-7.72 (m, 2H), 7.41-7.40 (m, 3H),

3.61 (t, 2H), 1.73-1.67 (m, 2H), 1.35-1.29 (m, 8H), 0.91-0.89 (m, 3H). MW:129.27.

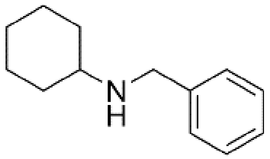


***N*-cyclohexyl-benzenemethanamine (1l).** $^{1}$H NMR (500 MHz, $CDCl_3$): δ = 7.35-7.26 (m, 5H), 3.82 (s, 1H), 2.54-2.48 (m, 1H), 1.88-1.10 (m, 10H). MW: 189.30.

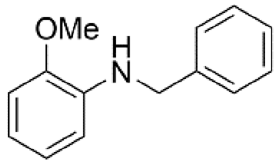


***N*-(2-methoxyphenyl)benzenemethanamine (1m).** $^{1}$H NMR (500 MHz, $CDCl_3$): δ = 7.40-7.26 (m, 5H), 6.85-6.77 (m, 2H), 6.69-6.63 (m, 2H), 6.55 (s, 1H), 4.34 (s, 2H), 3.81 (s, 3H). MW: 213.30.

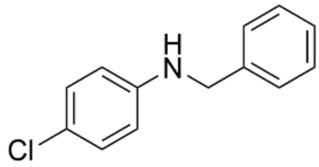


***N*-(4-chlorophenyl)benzenemethanamine (1n).** $^{1}$H NMR (500 MHz, $CDCl_3$): δ = 7.44-7.42 (m, 4H), 7.33 (m, 2H), 6.63 (m, 1H), 6.51 (d, 2H), 4.23 (s, 2H) 3.92 (s, 1H). MW: 216.70

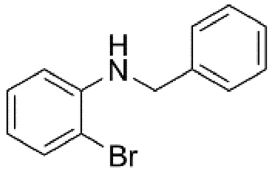


***N*-(2-bromophenyl)benzenemethanamine (1o).** $^{1}$H NMR (500 MHz, $CDCl_3$): δ = 7.43-7.39 (d, 1H), 7.33-7.22 (m, 5H), 7.13-7.08 (m, 1H), 6.58-6.52 (m, 2H), 4.63 (s, 1H), 4.33 (s, 2H). MW: 262.15

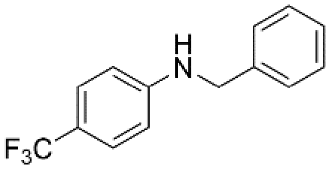


***N*-(4-trifluoromethylphenyl)benzenemethanamine (1p).** $^{1}$H NMR (500 MHz, $CDCl_3$): δ = 7.45-7.40 (m,

5H), 7.41-7.37 (m, 3H), 6.65 (d, 2H) 4.41 (s, 2H). MW: 251.26.

References:

1. Morris, W.; Briley, W. E.; Auyeung, E.; Cabezas, M. D.; Mirkin, C. A., *Journal of the American Chemical Society* **2014,** 136 (20), 7261-7264.
2. Wong, C. M.; McBurney, R. T.; Binding, S. C.; Peterson, M. B.; Gonçales, V. R.; Gooding, J. J.; Messerle, B. A., *Green Chemistry* **2017,** 19 (13), 3142-3151.
3. Choi, I.; Chun, S.; Chung, Y. K., *The Journal of Organic Chemistry* **2017,** 82 (23), 12771-12777.
4. Lan, X.-B.; Ye, Z.; Yang, C.; Li, W.; Liu, J.; Huang, M.; Liu, Y.; Ke, Z., *ChemSusChem* **2021,** 14 (3), 860-865.
